# Expert Perspectives on Transparency and Trust in AI-Based Clinical Decision Support Systems for Chest X-Ray Diagnosis: Qualitative Study

**DOI:** 10.2196/99472

**Published:** 2026-11-05

**Authors:** Yada Sriviboon, Arisara Jiamsanguanwong

**Affiliations:** 1 Department of Industrial Engineering, Center of Excellence for Human-Robot Collaboration and Systems Integration, Faculty of Engineering, Chulalongkorn University, 254 Phayathai Rd, Pathumwan, Bangkok, 10330, Thailand, +66 2218 6829

**Keywords:** transparency, trust, user interface, user’s mental model, AI-CDSS, AI-based Clinical Decision Support Systems

## Abstract

**Background:**

The integration of AI-based Clinical Decision Support Systems (AI-CDSS) into clinical practice has expanded rapidly, particularly in medical imaging. Beyond algorithmic performance, growing evidence suggests that clinicians’ engagement with AI-CDSS is shaped by how transparently systems communicate their outputs, and how trust is formed through human-AI interaction. However, a limited understanding of how these concepts are perceived by end users and developers continues to hinder real-world implementation.

**Objective:**

This study aims to explore expert perspectives on transparency and trust in AI-CDSS for chest X-ray diagnosis, with a focus on the two-dimensional lens: user interface and users’ mental models.

**Methods:**

A qualitative study was conducted using semistructured interviews with a total of 21 experts, including 11 radiologists and 10 AI experts experienced in AI-CDSS. Data were analyzed thematically to explore factors perceived to influence transparent communication and trust.

**Results:**

Three themes emerged: (1) perspective of transparency and trust, (2) user interface, and (3) users’ mental models. Findings reveal that trust is a dynamic calibration process developed through repeated use, feedback loops, and alignment with clinicians’ mental models, rather than algorithmic accuracy alone. Significant discrepancies were identified between how developers design system transparency and how clinicians interpret AI outputs.

**Conclusions:**

This study demonstrates that transparency and trust are achieved through interpretable outputs, clear communication of system limitations, and alignment with clinical workflows. Designing trustworthy AI-CDSS requires integrating transparent interface design with consideration of users’ knowledge, experience, and attitudes to support effective human-AI interaction.

## Introduction

AI-based Clinical Decision Support Systems (AI-CDSS) represent an advanced evolution of conventional decision support technologies, leveraging AI techniques to augment clinical decision-making processes. By using sophisticated algorithms such as machine learning and deep learning, AI-CDSS are capable of interpreting and analyzing large volumes of complex medical data, including radiological images, laboratory results, and electronic health records [1,2]. Rather than replacing medical professionals, these systems are designed to support the diagnostic process, assisting medical professionals in generating hypotheses, identifying patterns, and reducing the likelihood of diagnostic errors, thereby enhancing both the quality and efficiency of health care delivery [3,4].

Despite these technological advances, the successful integration of AI-CDSS into clinical practice depends not only on algorithmic performance but also on how effectively these systems communicate their outputs to medical professionals and support informed human-AI collaboration [5]. AI characteristics, such as lack of transparency, self-learning, and autonomous characteristics, bring uncertainty and pose significant challenges to establishing trust in the implementation of AI [6]. Transparency refers to the disclosure of relevant information about a system’s decision-making processes, procedures, performance, and functioning [7]. In this study, transparent communication denotes the system-user communication aspect of transparency, specifically making information about AI-CDSS understandable to clinicians so they can evaluate its outputs. Explainability concerns explanations that clarify a model’s internal functioning, whereas interpretability concerns the extent to which a model is understandable to a human [8]. A perceived lack of transparency in system design and operation has been shown to undermine users’ trust in digital interfaces and decision-support processes [9]. In the context of AI-CDSS, trust refers to the attitude that the system will help clinicians achieve their goals under conditions of uncertainty and vulnerability [10]. Trust in a specific technology can further be understood through users’ beliefs about its functionality, helpfulness, and reliability [11]. Trustworthiness refers to the properties of an AI system that justify placing trust in it [12]. Trust is distinct from reliance, which refers to the behavioral use of automation; trust guides, but does not fully determine, reliance [13]. In this study, trust calibration is used to describe whether clinicians’ trust is appropriately aligned with the system’s reliability [10].

When transparency is insufficient, medical professionals may struggle to understand how and on what basis AI systems generate recommendations, limiting their ability to evaluate, justify, or appropriately rely on AI outputs [14,15]. As a result, when users are unable to discern the rationale behind AI recommendations, concerns about the system’s effectiveness and the potential for patient harm are heightened among medical professionals [16]. In contrast, transparent AI systems can support informed clinical judgment by enabling users to critically assess AI recommendations while retaining professional autonomy in decision-making [17]. Nevertheless, adoption remains limited; recent reports indicate that 64% of medical professionals in the United States have not adopted AI technologies, with concerns about the credibility of AI-generated information cited as a primary barrier [18].

To examine transparent communication in context, this study adopts a conceptual lens grounded in human-computer interaction (HCI) and human factors, drawing on the concepts of the Gulf of Execution and Gulf of Evaluation [19] and Carroll and Olson's discussion of mental models in HCI [20]. These frameworks inform a two-dimensional perspective on human-AI interaction: the technology dimension, concerning how information is conveyed through the user interface, and the human dimension, focused on users’ mental models, which are an individual’s knowledge and understanding that form the basis for constructing reasonable actions and explanations about why certain actions are appropriate and predictive reasoning regarding both familiar and novel situations [21,22].

Understanding transparency in AI-CDSS requires insights from both system developers and clinical end users [23]. They may differ in their goals and interpretations of explainable AI, highlighting the value of considering both perspectives [24]. AI experts play a critical role in determining how systems are developed and which features and explanations are implemented and presented through the user interface. In contrast, medical professionals, as end users, must interpret AI outputs through their mental models and integrate them into clinical reasoning. Discrepancies between how transparency is designed and how it is perceived can lead to misalignment in human-AI interaction, resulting in inappropriate reliance on AI recommendations. Therefore, incorporating perspectives from both AI experts and medical professionals is essential for understanding how user interface design and user mental models may support transparency and trust in real-world clinical decision-making contexts.

Accordingly, this study aims to explore expert perspectives on transparency and trust in AI-CDSS for chest X-ray diagnosis through a qualitative, semistructured interview approach. This study addresses the following research questions (RQs):

- RQ1: How do medical professionals and AI experts perceive transparency and trust in AI-CDSS?
- RQ2: What user interface-related information and design elements are perceived by medical professionals and AI experts as important for supporting transparent communication and/or trust in AI-CDSS?
- RQ3: What aspects of users’ mental models are perceived by medical professionals and AI experts as important for supporting transparent communication and/or trust in AI-CDSS?

This study contributes to the literature on human-AI interaction in AI-CDSS in 2 key ways. First, it provides qualitative insights into what transparency and trust in AI-CDSS are perceived to mean from the perspectives of both AI experts and medical professionals, grounded in the context of chest X-ray diagnosis. Second, it elucidates what user interface design considerations and users’ mental models are perceived to support transparent communication and trust in AI-CDSS, providing qualitative insights to guide future design of AI-CDSS in real-world clinical settings.

## Methods

### Interview Design

This study used a qualitative research design using semistructured, one-to-one expert interviews to explore perceptions of transparency and trust in AI-CDSS for chest X-ray diagnosis. The interview approach was designed to elicit in-depth insights into user interface design considerations and users’ mental models perceived to support transparent communication and trust in AI-CDSS. One-to-one interviews were selected to encourage open discussion, enabling participants to articulate their perspectives based on professional experience.

All participants were asked to reflect on AI-CDSS for chest X-ray interpretation. No common scenario, AI-CDSS interface screenshot, or example AI output was presented; radiologists reflected on systems they had used, whereas AI experts referred to systems they had developed or worked with. The systems discussed were designed to detect or localize suspected abnormalities to assist clinicians in interpreting chest radiographs. Interview questions addressed user interface elements, including heatmaps, confidence information, and feedback mechanisms that allow users to report errors. The interview questions were developed from the research questions and a review of relevant literature on transparency, trust, user interface design, and users’ mental models in human-AI interaction. The guides began with questions about radiologists’ experience with AI-CDSS or AI experts’ goals in developing such systems, followed by questions on transparency and trust, user interface design, and users’ mental models. Questions were tailored to each group’s role: radiologists were asked about clinical use and interpretation, whereas AI experts were asked about development and design considerations. Open-ended questions invited explanations and additional suggestions beyond the specified topics. To ensure clarity, relevance, and content validity, the interview guide was reviewed by domain experts using the Index of Item-Objective Congruence (IOC). Following revision, all interview questions received an IOC score of 1.00. Before data collection, the guide was reviewed by a health care innovation expert and a physician who served as the head of a radiology center. However, it was not formally pilot tested or cognitively pretested with representatives of the target groups. The interview guide was not modified after data collection began. Interviews were conducted in Thai to facilitate the accurate expression of participants’ perspectives. The validated interview questions are provided in Multimedia Appendix 1.

### Expert Selection

Purposive sampling was used to recruit experts whose professional backgrounds aligned with the objectives of the study. Potential participants were identified based on their professional roles and experience in either the development or clinical use of AI-CDSS for chest X-ray diagnosis. Eligible experts were invited to participate via direct contact (eg, email or professional networks), and participation was entirely voluntary.

Two groups of experts were included to capture complementary perspectives: (1) Medical professionals specializing in radiology for at least 5 years, with at least 3 years of experience using AI-CDSS for chest X-ray in clinical diagnostic practice. (2) AI experts with a minimum of 5 years of experience in developing AI technologies for medical imaging. The selection criteria for experts in this study were established based on the minimum threshold of 5 years of professional experience in their respective fields. This duration ensures that participants have surpassed the initial skill acquisition stages and possess the necessary practical wisdom to provide insightful perspectives [25,26]. For medical professionals, a minimum of 3 years of experience using AI-CDSS for chest X-ray was required, as the widespread adoption of AI-CDSS in clinical practice in Thailand has occurred only in recent years, beginning around 2021. The sample size for the qualitative interviews was guided by recommendations in qualitative research, suggesting that between 5 and 30 participants are generally sufficient for in-depth interviews, depending on the complexity of the research questions. Recruitment continued until theoretical saturation was achieved, that is, when additional interviews no longer yielded new insights relevant to the RQs [27].

### Procedure

Qualitative data were collected through online interviews conducted via Microsoft Teams. Before the interviews, participants received an informed consent form detailing the study objectives, procedures, and ethical considerations, followed by a briefing using Thai-language slides covering the study objectives, interview structure, key concepts, and participants’ rights. Participants then completed a brief questionnaire collecting demographic and professional background information before their interviews began. The briefing slides and their English translation are provided in Multimedia Appendix 2. Separate, but conceptually aligned interview guides were used for AI experts and radiologists. Interviews typically lasted 20‐30 minutes. All interviews were audio-recorded with participants’ informed consent and transcribed verbatim for subsequent analysis.

### Data Analysis

Interview transcripts were analyzed using thematic analysis following the six-phase approach proposed by Braun and Clarke [28]. This method was selected for its suitability in identifying patterned meanings across expert accounts while remaining grounded in participants’ perspectives and the study’s RQs. Analysis began with familiarization through repeated reading of transcripts to gain an overall understanding of participants’ views. Initial codes were then generated inductively, focusing on statements related to transparency and trust, user interface, and users’ mental models in AI-CDSS use. Coding was conducted across both expert groups (radiologists and AI experts) to identify shared and differing perspectives. Next, codes were examined and grouped into potential themes by identifying conceptual relationships and recurring patterns across participants. These candidate themes were iteratively reviewed and refined to ensure internal coherence and clear distinction between themes. Finally, themes were reported with illustrative quotations to preserve participants’ voices and to demonstrate analytic grounding in the data. The quotations selected for reporting were translated from Thai into English by the principal investigator. The English translations were subsequently checked against the original Thai quotations in collaboration with coresearcher (AJ) to ensure accuracy and preserve the intended meaning.

### Ethical Considerations

The study was approved by the Research Ethics Review Committee for Research Involving Human Research Participants, Group I, Chulalongkorn University, Thailand (Certificate of Approval number 079/69). The protocol adhered to the Belmont Report (1979), Declaration of Helsinki (2013), Council for International Organizations of Medical Sciences (2016), Standards of Research Ethics Committee (2017), and National Policy for Human Research (2015).

## Results

### Overview

A total of 21 experts participated in the study, including 11 radiologists and 10 AI experts. Among the radiologists, 10 of 11 (91%) participants were female, and 1 of 11 (9%) participants was male. In terms of workplace, 7 of 11 (64%) participants worked in private hospitals, and 4 of 11 (36%) participants worked in public hospitals. Participants had substantial clinical experience, ranging from 5 to 33 years, with a mean of 16.0 (SD 8.94) years of clinical practice in radiology. All radiologists had direct experience using AI-CDSS for chest X-ray diagnosis, with a mean AI-CDSS usage experience of 4 (SD 0) years. On average, participants interpreted approximately 30.9 (SD 8.31) chest X-ray cases per day, reflecting routine engagement with high-volume diagnostic tasks. Among the AI experts, 3 of 10 (30%) participants were female, and 7 of 10 (70%) participants were male, with professional roles spanning professor, associate professor, assistant professor, lecturer, researcher, and chief technology officer (CTO). In terms of affiliation, 8 of 10 (80%) participants were affiliated with public universities, 1 of 10 (10%) participants was affiliated with a private university, and 1 of 10 (10%) participants was affiliated with an AI-based medical imaging company. The AI experts had a mean of 9.4 (SD 3.5) years of experience in AI and medical imaging–related research or development, ensuring in-depth familiarity with model development, explainability techniques, and user interface design for chest X-ray applications. Participant characteristics for radiologists are presented in Table 1, and for AI experts in Table 2.

**Table 1.** Characteristics of radiologist participants (n=11).

ID	Gender	Years in radiology	AI-CDSS^a^ experience (years)	Hospital type	Frequency of AI-CDSS use
R1	Female	24	4	Private	Daily
R2	Female	33	4	Private	Daily
R3	Female	18	4	Private	Daily
R4	Female	28	4	Private	Daily
R5	Female	14	4	Private	Daily
R6	Female	14	4	Private	Daily
R7	Female	12	4	Public	Daily
R8	Female	7	4	Public	Daily
R9	Female	8	4	Private	Daily
R10	Male	5	4	Public	Daily
R11	Female	13	4	Public	Daily

a AI-CDSS: AI–based Clinical Decision Support Systems.

**Table 2.** Characteristics of AI expert participants (n=10).

ID	Gender	Years in AI (medical imaging)	Highest degree	Affiliation type	Role/position
A1	Female	10	PhD	Public university	Associate professor
A2	Male	6	MD	Public university	Associate professor
A3	Female	8	PhD	Public university	Lecturer
A4	Female	8	PhD	Public university	Assistant professor
A5	Male	14	PhD	Public university	Associate professor
A6	Male	9	PhD	Public university	Assistant professor
A7	Male	15	PhD	Private university	Professor
A8	Male	13	PhD	Public university	Associate professor
A9	Male	6	MD	AI-based medical imaging company	CTO^a^
A10	Male	5	MD	Public university	Researcher

a CTO: chief technology officer.

The results use a participant coding scheme to attribute quotes and insights while preserving confidentiality. Each participant is identified using a shorthand code (X, Y, and Z), where X denotes the participant’s role (Radiologist [R] or AI expert [A]), Y indicates gender (Male [M] or Female [F]), and Z represents years of professional experience in radiology or AI development in health care. A total of 266 attributed quotations were identified across the interview dataset. Table 3 presents their distribution across the 3 themes and associated subthemes, together with sample quotations. The first theme, Perspective of transparency and trust, describes participants’ perspectives on transparency and trust when interacting with the AI-CDSS. The second theme, User Interface, highlights how specific user interface design elements facilitate clear communication and foster trust in the AI’s recommendations. The third theme, Users’ Mental Models, illustrates how participants’ experience, attitude, and knowledge about AI shape their interpretation of the AI’s outputs and their trust in the system. Figure 1 presents an overview of the 3 themes, their associated subthemes, and the key findings within each subtheme.

**Table 3.** Distribution of attributed quotations across the themes and subthemes, with sample quotes.

Themes and subthemes, n (%)^a^	Sample quotes
Perspective of^b^ transparency and trust	
Transparency that clinicians need (n=24, 9%)	“Radiologists don’t expect to understand the whole model, but they need to know why it flags something.” (A8, M, 13) “Heatmap shows what the AI is looking at.” (A7, M, 15)
Perceived role of AI (n=29, 10.9%)	“AI is like a friend double-checking for me.” (R3, F, 18) “If it flags something I didn’t see, I’ll recheck that spot.” (R7, F, 12)
Trust calibration (n=19,7.1%)	“I trust it more when it shows confidence levels.” (R5, F, 14) “We know which findings it’s good or weak at.” (R1, F, 24) “Users need to know what AI can and can’t do.”(A5, M, 14)
User interface^c^	
What information should be presented (n=42,15.8%)	“Heatmap and confidence levels are most useful.” (R9, F, 8) “If AI can’t give context,. it may mislead on TB.” (R8, F, 7) “Radiologists wanted AI to prioritize urgent abnormalities for quicker clinical action.” (R5, F, 14) “Developers should provide details about the training data. what diseases it learned from.” (A2, M, 6)
How should information be presented (n=27, 10.2%)	“I prefer information shown as images rather than text. Numbers are okay, but not too much.” (A1, F, 10) “Don’t clutter the screen with too much info.” (R6, F, 14)
Feedback loop (n=14, 5.3%)	“Feedback is a must; otherwise, errors persist.” (A8, M, 13)
Users’ mental models^d^	
Knowledge (n=54, 20.3%)	“You don’t need to understand deep algorithms, just where the answers come from.” (R1, F, 24) “We need enough knowledge to use the system and interpret the results.” (A5, M, 14)
Experience (n=24, 9.0%)	“We’ve learned what it often gets wrong and check those again.” (R9, F, 8) “Clinical experience and years in practice matter. For example, newly graduated doctors are more likely to trust the AI outputs without question.” (R4, F, 28) “If doctors are involved from the start, they understand the AI better.” (A2, M, 6)
Attitude (n=33, 12.4%)	“Younger doctors are more likely to use and trust the system because they grew up with technology.” (R9, F, 8) “Doctors must be open to using it or they’ll fall behind.” (R10, M, 5)

a n represents attributed quotations and % represents % of total quotations.

b Theme total, n (%): 72 (27.1).

c Theme total, n (%): 83 (31.2).

d Theme total, n (%): 111 (41.7).

**Figure 1.**
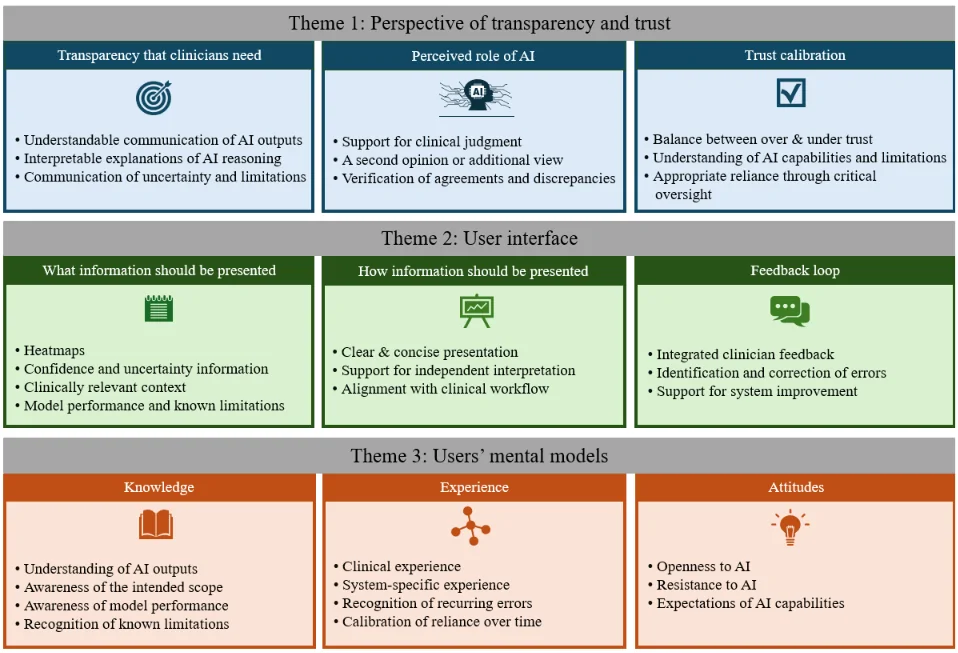
An overview of the 3 themes, their associated subthemes, and the key findings within each subtheme.

### Theme 1: Perspective of Transparency and Trust

#### Overview

The perspective of transparency and trust in AI-CDSS was structured into 3 interrelated subthemes: Transparency That Clinicians Need, Perceived Role of AI, and Trust Calibration. These subthemes reflect how both radiologists and AI developers conceptualized the meaning and importance of transparency, the functional role of AI in clinical practice, and the dynamic process of trust formation.

#### Transparency That Clinicians Need

Radiologists and AI experts converged on the view that transparency in AI-CDSS refers not to full algorithmic interpretability, but to the system’s ability to communicate its reasoning and limitations in a manner that radiologists can meaningfully engage with. This form of functional transparency was widely seen as foundational to trust. One AI expert distinguished model transparency, concerning access to the system’s internal logic, from information transparency, concerning the understandable communication of its outputs.

Participants from both groups emphasized that without visual or numerical explanations, such as heatmaps or confidence levels, AI-CDSS systems risk becoming “black boxes” and alienating their users. Participants described these explanations as helping clinicians understand:

What the AI is looking at when generating findings.

[A7, M, 15 years of experience]

Highlighted regions, confidence scores, and system rationales were viewed as interpretable cues that enable clinicians to scrutinize and contextualize AI outputs. As one AI expert remarked:

Radiologists don’t expect to understand the whole model, but they need to know why it flags something; that’s what explanation means to them.

[A8, M, 13 years of experience]

Turning to trust, both groups emphasized that it must be developed over time through consistent performance and transparent communication. While transparency facilitated trust formation, accuracy was seen as its core foundation:

Trust [in the AI] depends on its accuracy. If I know it’s very accurate on a particular finding, I tend to trust it.

[R1, F, 24 years of experience]

Explanations aligned with clinical reasoning were also perceived to enhance the system’s trustworthiness:

Providing an explanation makes it look more trustworthy and might make me trust it more.

[R5, F, 14 years of experience]

#### Perceived Role of AI

Participants from both groups described AI-CDSS as a supportive tool that complements but does not replace clinical judgment. Rather than perceiving the system as authoritative, radiologists framed it as a “second opinion” or “additional view” that helps validate or challenge their initial impression. As one participant remarked, using the system was “like playing a spot-the-difference game” (R3, F, 18) where AI cues helped direct attention to potential oversights.

Several radiologists described a consistent 4-step process: (1) reviewing the original radiograph, (2) forming a preliminary diagnosis, (3) consulting the AI’s output, and (4) revisiting the film to compare areas of agreement or discrepancy. Agreement reinforced their initial interpretation, whereas unexpected findings prompted further examination rather than automatic acceptance or dismissal. Radiologists described the AI-CDSS as a useful double-check mechanism to either confirm their own assessments or draw attention to possible oversights:

AI is like another friend helping me diagnose. If it flags the same area which I’m concerned about, then we’re done. If it flags something different, I’ll investigate that spot further.

[R3, F, 18 years of experience]

#### Trust Calibration

Several AI experts emphasized that system design alone cannot ensure appropriate trust. Rather, users’ mental models, their understanding of what the AI can and cannot do, play a critical role:

Achieving the right level of trust comes down to communication and AI literacy… The system alone can’t fix it; we need to talk to users about what the AI can and cannot do.

[A6, M, 9 years of experience]

Importantly, trust was not viewed as binary. Instead, experts described a need for trust calibration, where overtrust may lead to uncritical acceptance, and undertrust may result in dismissal of potentially helpful insights. Most participants believed undertrust to be more prevalent in practice:

Physicians are quite cautious, so under-trust happens more than over-trust in practice.

[A5, M, 14 years of experience]

### Theme 2: User Interface

#### Overview

Participants’ perspectives on user interface design were organized around 3 interrelated subthemes: what information the system should present to users, how that information should be presented, and the feedback loop. Across these subthemes, both radiologists and AI experts emphasized the importance of clarity, intuitiveness, and alignment with clinical workflows, although their priorities differed according to their professional roles.

#### What Information Should Be Presented

Participants in both groups identified specific types of information that they considered important for an AI-CDSS to present to support transparency. For radiologists, the most essential features were the visual heatmap, highlighting areas of suspected abnormality, and the confidence level associated with each finding. One radiologist explained:

The heatmap shows where AI is suspicious, how much it’s suspicious in each spot, which helps boost my confidence, because I can see what it saw.

[R10, M, 5 years of experience]

Radiologists described heatmaps as helping them localize and evaluate AI-identified findings, whereas confidence information indicated the strength of each prediction. Although both were considered foundational, radiologists varied in the importance they assigned to each element. For instance:

I’m mainly interested in the heatmap; the confidence level is only a little.

[R11, F, 13 years of experience]

Some radiologists also requested richer textual or visual explanations that communicated not only where an abnormality was detected but also why the AI produced its conclusion.

I’d trust it more if it could explain how it got to its answer. That would make a real difference.

[R6, F, 14 years of experience]

Suggestions included distinguishing between types of detected lesions (eg, calcified nodule vs soft-tissue mass) and providing context-sensitive reasoning. Particularly for findings that typically require patient history, like tuberculosis, radiologists cautioned that AI predictions without context could lead to overdiagnosis or false reassurance:

If the AI can’t account for clinical context, it might label something as TB and be misleading, which many of us find inaccurate.

[R8, F, 7 years of experience]

One participant recommended that such ambiguous outputs be either suppressed or clearly flagged as uncertain to prevent misinterpretation. Conversely, urgent and actionable abnormalities, such as pneumothorax, could be prioritized to support timely clinical action. Radiologists wanted information on how confidence scores were calculated, alongside broader performance measures such as sensitivity, specificity, and known limitations. As one noted:

Approximately how accurate is this model, and how much can I trust it? I want to know the range of what it’s good and not good at.

[R5, F, 14 years of experience]

AI experts echoed many of these needs but proposed additional forms of information. In addition to heatmaps and scores, they suggested providing textual explanations that resemble mini radiology reports, generated via explainable or generative AI. For instance:

The AI sees a diffuse haziness in the right lung, suggestive of pneumonia, because the opacity has XXX characteristics.

[A3, F, 8 years of experience]

They emphasized the importance of model-centric transparency, such as detailing training data characteristics, model provenance, and blind spots. AI experts believed such background would help users understand why the AI behaves the way it does:

Developers should provide details about the training data, what diseases it learned from, and how balanced it was.

[A2, M, 6 years of experience]

Another area of emphasis was flagging uncertainty. AI experts suggested interface features for indicating low-confidence predictions (eg, color-coded warnings or disclaimers), allowing users to calibrate their trust accordingly. Conversely, high-confidence findings could be visually marked to reassure users:

If a prediction is borderline, the system should say so clearly. We don’t want users to over-trust it by default.

[A4, F, 8 years of experience]

Overall, both groups regarded heatmaps and confidence levels as foundational to transparency, although radiologists emphasized actionable information, prioritization of urgent findings, and avoidance of misleading content, whereas AI developers emphasized broader system transparency, explanation depth, and interface adaptability.

#### How Should Information Be Presented

Participants from both groups emphasized that AI outputs should be clinically usable: easy to perceive, quick to interpret, and aligned with real diagnostic workflows, while avoiding interface burden or cognitive overload. Radiologists particularly preferred control over when AI outputs became visible. Many wanted AI outputs to function as a secondary layer rather than being imposed upfront, allowing clinicians first to form an independent impression and then consult AI for confirmation or discrepancy-checking. This was reflected in requests for toggle-based interactions, where heatmaps can be switched on and off over the original image:

The heatmap could be shown by clicking once to overlay it on the original, like turning it on and off; however, it depends on the hospital settings.

[R1, F, 24 years of experience]

Radiologists also emphasized that AI-marked images should maintain visual comparability with standard reads:

The AI image should be the same size as the original film so doctors can clearly see the point the AI is indicating.

[R1, F, 24 years of experience]

Participants from both groups also emphasized managing information density and screen clutter, which they associated with cognitive load and decision time. Radiologists cautioned against overcrowded interfaces:

Be careful not to have too much information until the screen becomes messy.

[R6, F, 14 years of experience]

Radiologists also regarded excessive textual information as problematic and expressed irritation when interfaces fragmented attention unnecessarily, such as separating outputs into multiple views for minor findings:

If it splits out heatmaps for every finding, especially for tiny things, it becomes annoying.”

[R7, F, 12 years of experience]

Another recurring concern involved clear visual mapping between highlighted regions and diagnostic meaning, particularly when multiple findings are present. Radiologists requested that heatmaps explicitly indicate which abnormality each region corresponds to:

I want the heatmap to clearly indicate which lesion it belongs to. If it’s combined into one image, it should identify the location.

[R10, M, 5 years of experience]

From the AI experts’ perspective, these concerns aligned with a preference for visual rather than text-heavy presentation:

I prefer information shown as images rather than text. Numbers are okay, but not too much.

[A1, F, 10 years of experience]

AI experts additionally emphasized that presentation requirements are task-dependent, cautioning against one-size-fits-all visualization strategies:

How to show the heatmap depends more on the task. For COVID, we look at the overall lung pattern, not a single point, but for some diseases, you need segmentation or boundary boxes.

[A6, M, 9 years of experience]

They also stressed the importance of involving clinicians early in interface design to avoid cumulative microburdens:

Doctors should be involved from the beginning. Even one extra click is not okay if they do the task 100 times, that’s 100 extra clicks.

[A5, M, 14 years of experience]

Relatedly, experts highlighted that interface familiarity and prioritization matter:

Design should ask doctors as the main users about what they want to see first, second, third. Even the old interface style matters because it links to user experience.

[A6, M, 9 years of experience]

Several AI experts described desirable enhancements in interaction and fidelity, while still adhering to a minimalist philosophy. For example, interactive image manipulation was suggested:

Ideally, the image should be interactive, and the user can rotate it and view it from different angles.

[A1, F, 10 years of experience]

Others emphasized that AI displays should not disrupt ongoing clinical work:

If original and AI images can be placed side by side or switched between modes easily, the key is that it must not interfere with the physician’s workflow.

[A8, M, 13 years of experience]

Some experts advocated structuring heatmaps per abnormality to preserve interpretability. At the same time, restraint remained a shared design value:

Less is more. Don’t make it complicated, provide enough information for decision-making.

[A9, M, 6 years of experience]

Overall, radiologists emphasized workflow protection and low-clutter clarity, whereas AI experts emphasized task-dependent visualization, clinician co-design, and interaction design that minimizes cumulative burden. Both groups emphasized presenting sufficient information for clinical decision-making without disrupting workflow.

#### Feedback Loop

Participants also described transparency as an interactive process that should enable users to respond to AI outputs, identify errors, and contribute feedback for system improvement. Both radiologists and AI experts emphasized that feedback loops are essential for sustaining trust, calibrating reliance, and improving real-world performance. Radiologists consistently framed feedback as a “must-have” feature rather than an optional add-on, particularly because clinicians are the first to notice recurring errors or shifts in system behavior.

Radiologists emphasized the need for direct, integrated feedback mechanisms that would allow them to mark AI outputs as correct or incorrect, identify error locations, and optionally add brief comments. These features were considered especially important for recurrent false positives, such as nipple shadows, electrocardiogram (ECG) leads, or bone artifacts, which clinicians can easily recognize but which repeatedly undermine confidence when misclassified:

If the AI keeps misreading the same obvious things, it feels unreliable. Being able to flag those mistakes would really help.

[R3, F, 18 years of experience]

Participants emphasized that feedback should be simple, efficient, and integrated into the reading workflow, as indirect reporting may delay system improvement and reduce clinicians’ sense of agency.

AI experts further stressed that the depth and quality of feedback matter. Superficial signals alone (eg, binary correct/incorrect) were considered insufficient, whereas expert-level feedback, such as identifying error types, anatomical regions, or contextual causes, was seen as substantially more valuable for refining model behavior:

Feedback from experienced doctors is especially valuable because they can comment at a deeper level, not just right or wrong.

[A1, F, 10 years of experience]

Both groups cautioned that feedback mechanisms must be carefully designed so as not to burden clinicians or disrupt clinical workflows. AI experts emphasized that even small increases in interaction cost could quickly become unacceptable in high-volume practice:

We want feedback from clinicians every time they use the system, but it has to be effortless. The interaction must be simple, intuitive, and require as little movement and clicking as possible; otherwise, it won’t be used in real practice.

[A5, M, 14 years of experience]

Overall, both groups regarded feedback as an interactive component of transparency but differed in emphasis. Radiologists emphasized its role in reporting recurring errors and maintaining an active role in AI use, whereas AI experts highlighted the importance of obtaining clinically meaningful feedback through low-burden mechanisms.

### Theme 3: Users’ Mental Models

#### Overview

Participants’ accounts indicated that transparent communication in AI-CDSS is also influenced by users’ mental models of the system. In this study, this theme comprises the following subthemes: knowledge, experience, and attitudes. Within this theme, radiologists described the knowledge, experience, and attitudes shaping their interaction with AI-CDSS, whereas AI experts provided development-oriented observations about how these user characteristics may affect system interpretation and use.

#### Knowledge

User knowledge emerged as a foundational factor for transparent and appropriate interaction with AI-CDSS. Participants from both groups framed the knowledge required for AI-CDSS use not as deep technical expertise in algorithms, but as a conceptual understanding of how the system generates output, what it is trained to detect, and where its limitations lie. Radiologists emphasized that effective AI use presupposes sufficient domain knowledge. AI-CDSS was repeatedly described as an assistive tool that relies on the clinician’s ability to judge plausibility, recognize artifacts, and identify common pitfalls:

You don’t need to understand deep algorithms, just where the answers come from. If doctors know that, they won’t question every AI output unnecessarily.

[R1, F, 24 years of experience]

Doctors must know enough to check whether the AI is right or wrong. When it flags something, I try to understand why it appears.

[R5, F, 14 years of experience]

Radiologists described their clinical knowledge as helping them recognize likely false positives arising from normal anatomy or artifacts, such as calcifications, vessels, or external devices:

Because we’ve been trained, we can already guess where false positives are likely to occur.

[R10, M, 5 years of experience]

Beyond clinical expertise, participants highlighted the importance of understanding the AI system itself, particularly its scope, training focus, and performance variability across findings. Radiologists did not expect to understand model internals but wanted clarity on what the system was trained to detect, where it performed reliably, and where errors were likely:

If you know the sensitivity and specificity, you can decide how much to trust it in different situations.

[R9, F, 8 years of experience]

Participants associated such knowledge with decisions about when to rely on or further verify AI outputs:

Knowing the strengths and limitations helps doctors plan their work, where to double-check more and where they can rely on it.”

[A2, M, 6 years of experience]

Participants also stressed the need to correctly interpret system outputs, particularly confidence scores and visual explanations. AI experts cautioned against misinterpreting these parameters:

Doctors need to understand what confidence percentage really means. A confidence score doesn’t guarantee the heatmap location is correct.

[A10, M, 5 years of experience]

Training was considered necessary, with both groups recommending structured training covering system use, interpretation, and limitations:

Every time new software is installed, there must be training, both an overview and how to use it in practice.

[A5, M, 14 years of experience]

AI experts emphasized that explaining validation logic, data characteristics, and experimental design without excessive technical detail helps users form realistic expectations:

We don’t explain deep techniques, but we explain how the experiments were designed and how the model was validated.

[A2, M, 6 years of experience]

Participants further noted that clinicians involved early in system development naturally developed stronger users’ mental models of the AI, while other users required clear manuals explaining displayed metrics and high-risk findings:

If doctors weren’t involved in development, they need a user manual explaining what each value means and which findings require caution.

[A4, F, 8 years of experience]

Overall, participants regarded clinical and system-specific knowledge as a basis for trust calibration. Training, expectation-setting, and clear documentation were considered necessary to help clinicians interpret AI outputs appropriately and understand the system’s scope and limitations.

#### Experience

Clinical experience and hands-on exposure to AI-CDSS shaped how radiologists incorporated AI outputs into their reading practice. Participants from both groups described a gradient in use patterns: less-experienced readers tended to rely on AI more readily, whereas highly experienced radiologists perceived fewer incremental benefits and primarily used AI for confirmation rather than guidance.

Experience with the AI tool itself was described as cumulative. With continued use, radiologists learned the system’s typical error patterns and adjusted their verification strategies, accordingly, scrutinizing findings known to be error-prone while moving more efficiently through scenarios where the AI performed reliably:

As we use it more, we learn where it often gets things wrong; those areas or findings we recheck more carefully, and we may look for additional details.”

[R8, F, 7 years of experience]

Radiologists described developing practical routines for when to consult AI and how much weight to assign to its outputs. Readiness to adopt AI was reported across experience levels, although reliance patterns differed. Many radiologists indicated that repeated exposure to real cases helped them calibrate their expectations and integrate AI into routine workflows:

If you keep using it, you learn and adapt with the AI; over time, you become more accepting and can balance how you use it.

[R6, F, 14 years of experience]

AI experts also noted that radiologists’ routine engagement with digital tools could facilitate AI integration:

Radiologists are already used to using new tools, so adoption is generally easier compared with some other fields.

[A2, M, 6 years of experience]

Overall, clinical and system-specific experience shaped when, how, and to what extent radiologists relied on AI-CDSS in diagnostic decision-making.

#### Attitudes

Participants from both groups described attitudes toward AI-CDSS as ranging from active resistance to pragmatic acceptance. Across interviews, attitudes were shaped by (1) beliefs about human-AI capability, (2) willingness to engage with new technology, and (3) perceived consequences of agreeing with or challenging AI outputs:

Some clinicians hold an ego mindset that AI can never be better than them, so they avoid using it or ignore the AI’s outputs.

[R1, F, 24 years of experience]

Another radiologist noted:

My own confidence in reading films affects how much I trust and believe in the AI.

[R11, F, 13 years of experience]

Radiologists regarded openness as important for adoption and for learning how to work with the system over time. They described this openness as an adaptive stance involving acceptance of AI as part of current practice rather than treating it as optional:

You must be open to using it; if you resist, you’ll fall behind. We need to move with it and use it to stay ahead.

[R10, M, 5 years of experience]

At the same time, AI experts also noted attitudinal extremes in trust calibration. Some clinicians were described as trusting immediately, while others continued to distrust it:

If they trust, they trust completely; if they distrust, they distrust completely.”

[A9, M, 6 years of experience]

Several radiologists also noted that different responses to AI could arise for nontechnical reasons such as reluctance to contradict the system, while others were perceived as seeing little need for AI because of strong confidence in their own judgment:

Some doctors believe in AI because they’re afraid to argue with it if AI says it’s right, they accept it even when it’s wrong. But doctors who strongly trust themselves, especially seniors, may see no need for it.

[R6, F, 14 years of experience]

AI experts emphasized that negative attitudes were often driven less by the idea of AI itself than by perceived burden or workflow disruption; clinicians were reported to reject systems that add steps or friction, regardless of model performance:

Most of the time, it’s not over-trust people don’t accept it because they see it as a burden and extra steps. If it adds steps, doctors won’t use it.

[A8, M, 13 years of experience]

Overall, attitudes toward AI-CDSS varied from openness to resistance and influenced how clinicians engaged with, interpreted, and acted on AI outputs.

## Discussion

### Transparency for Trust Calibration

Across the themes, our findings suggest that in AI-CDSS for chest X-ray, transparency is interpreted functionally, not as full access to model internals, but as the system’s ability to communicate why it flagged a finding, how certain it is, and where it is likely to fail in a way clinicians can meaningfully use. Its contribution to trust therefore depends not only on the information presented through the interface but also on clinicians’ knowledge, experience, and attitudes when evaluating that information. This interpretation is consistent with a recent systematic review that identified system transparency, interpretability, clinical reliability, and alignment with clinical judgment as factors shaping health care professionals’ trust in AI-CDSS [29]. In this context, when outputs are delivered as unexplained “answers,” users are pushed into accept/reject behavior without defensible justification, which can erode confidence over time and inhibit sustained adoption [30]. Our findings extend prior work by showing that trust develops dynamically through repeated comparisons between AI outputs and clinicians’ own interpretations, rather than from accuracy or transparency alone. In chest X-ray reading, radiologists described forming an independent judgment, consulting AI as a second opinion, and revisiting discordant findings, illustrating how transparency is integrated into clinical reasoning.

A key implication is that transparency should be designed to support trust calibration, not to maximize trust. In safety-critical domains, both extremes are harmful: undertrust produces disuse of potentially valuable support, while overtrust increases susceptibility to automation bias and uncritical acceptance. Recent commentary in clinical AI highlights automation bias as a realistic risk when users defer to AI recommendations despite uncertainty or imperfect reliability, especially under time pressure [31].

Accordingly, transparency mechanisms should be explicitly “calibration-facing”: they should (1) communicate uncertainty, (2) disclose known limitations and boundary conditions, and (3) provide performance expectations that are understandable and clinically relevant. These cues should support clinicians’ judgments about when to rely on, verify, or override AI outputs, rather than serve merely as signals of credibility. To address undertrust specifically, clear information about performance in relevant clinical tasks, together with training using representative cases, may help clinicians calibrate their trust to the system’s demonstrated reliability and recognize situations in which AI is likely to provide valuable support. When an AI output conflicts with an initial interpretation, the user interface could provide a neutral prompt to reexamine the relevant region rather than a persuasive recommendation. This approach may discourage premature rejection without encouraging automatic acceptance. Clinician-facing transparency may also have implications for patient communication. Understanding the basis and limitations of AI outputs may help clinicians explain how AI contributed to a diagnosis and discuss uncertainty during shared decision-making. Such communication may influence patient trust and acceptance of AI-supported diagnoses, although these patient-facing implications were not examined in this study.

### Aligning Transparency With Clinical Decision-Making

Supporting trust calibration requires more than making system information available: clinicians must be able to interpret and use it within their workflow. Considered together, the user interface and users’ mental model findings suggest that transparency depends on aligning what the system communicates with users’ understanding and clinical tasks. A review of AI in radiology similarly emphasizes that integration success depends not only on performance but also on workflow fit and adoption factors; the “right” information must arrive in a form that clinicians will actually use at scale [32,33]. The radiology user interface literature has reported mixed findings regarding preferred interfaces: some users favor detailed displays combining heatmaps, text, and confidence information, whereas others prefer simple and uncomplicated designs [34]. The current study helps explain this apparent inconsistency. Participants did not necessarily prefer either more or less information in absolute terms; rather, they wanted information to be task-dependent, clinically relevant, and accessible without creating screen clutter or disrupting independent image interpretation. Within these shared requirements, radiologists emphasized actionable, context-sensitive cues, whereas AI experts additionally emphasized system-level information about training data, provenance, and limitations. These competing requirements may be partly reconciled through progressive disclosure, whereby essential information is presented initially, and further details are made available on demand [33,35]. In practice, localized findings and concise confidence or uncertainty information could appear on the primary display, while technical details about training data, validation, performance, and limitations could be accessed through secondary views. For critical abnormalities such as pneumothorax, the suspected finding, location, urgency, and uncertainty should be immediately visible to support timely verification and prioritization, whereas routine screening may benefit from a concise initial display with additional information available on demand to minimize workflow burden. Moreover, UI design requires more than simply displaying additional information; it must ensure that users can correctly interpret what each element represents. For example, in usability testing of a chest X-ray AI tool, radiologists were uncertain about how to interpret confidence values and requested guidance on which confidence levels should be considered clinically meaningful [36]. Additionally, AI assistance increased total reporting time by 14 (33%) seconds, although this difference did not reach statistical significance, with the additional decision-making time largely attributed to reviewing heatmaps [36]. These findings highlight a potential trade-off: additional explanations may support interpretability but can also increase cognitive and workflow demands without necessarily improving diagnostic performance. User interface elements should therefore be evaluated according to whether their benefits for understanding and clinical decision-making justify the additional time required. Beyond these usability considerations, the explanation and uncertainty methods themselves have important technical limitations. Current visual explanation methods, including saliency- and attribution-based heatmaps, may produce plausible-looking outputs without reliably explaining the basis or correctness of an individual prediction [37]. In evaluations using chest radiographs, saliency-map methods have also demonstrated limitations in localization utility, sensitivity to model randomization, repeatability, and reproducibility [38]. Similarly, predictive uncertainty estimates may become less reliable when deployment data differ from the training distribution [39]. Heatmaps and uncertainty estimates should therefore be presented as decision-support cues rather than definitive evidence of diagnostic correctness, together with clear information about their intended meaning, validation, and limitations.

Additionally, the user interface should include guardrails that reduce automation bias. Visual cues can improve efficiency, but they can also become persuasive anchors if not paired with uncertainty and boundary messaging. The automation-bias literature in clinical decision support underscores that systems should avoid presenting outputs in ways that encourage default deference; instead, they should support deliberate verification, especially for borderline or high-impact decisions [31]. Consistent with this concern, our findings suggest communicating uncertainty and limitations and presenting the original radiograph before introducing the AI output. For borderline outputs, additional guardrails could include explicit labels such as “uncertain” or “verification required,” avoidance of definitive recommendations, and concise prompts for independent verification. Under time pressure, such guardrails should be visually salient and limited to ambiguous or clinically consequential findings to facilitate verification without producing excessive alerts or workflow burden.

Finally, feedback extends this relationship beyond the interpretation of individual outputs by connecting clinicians’ recognition of errors with opportunities for system improvement. The findings suggest that allowing users to flag errors may also support agency and continued critical engagement with AI. This aligns with implementation recommendations that emphasize feedback loops as part of integrating AI into real-world clinical workflows, rather than treating deployment as a one-time event [32]. However, the presence of a feedback function alone is insufficient; it must be intuitive and integrated into the existing workflow while minimizing cognitive and interaction burden. Our findings revealed different priorities: radiologists emphasized immediate, workflow-integrated feedback, whereas AI experts valued detailed input but cautioned against workflow disruption. These priorities could be reconciled by embedding one-click confirmation or rejection directly into the image-reading interface while making error localization and brief comments optional. This approach would enable rapid routine feedback and more detailed input when needed without requiring a separate reporting process.

### Users’ Mental Models and the Interpretation of Transparency

The relationship between interface design and trust calibration depends partly on users’ mental models. This research suggests that the same transparency feature may be interpreted differently depending on clinicians’ knowledge, experience, and attitudes. This helps explain why “more explanation” is not automatically better: transparency is only effective when users can correctly interpret it and integrate it into clinical reasoning.

Previous evidence regarding the need for user training has been mixed. A recent scoping review reported that one included study characterized a chest X-ray AI system as easy to understand without requiring specialized training, although the evaluation used no formal scoring system and reported only limited qualitative findings [34]. By contrast, usability testing of another chest X-ray AI tool found that radiologists were uncertain about how to interpret confidence values and considered some form of training necessary before deployment [36]. More broadly, a systematic review identified training and familiarity as factors shaping trust and noted that opaque AI outputs may cause confusion and, in some cases, contribute to overreliance, particularly among less experienced clinicians [29]. Based on our findings, the 2 groups emphasized complementary pathways for developing users’ mental models. AI experts focused on structured information about system scope, validation, performance, and limitations, whereas radiologists described refining their understanding through repeated use and recognition of recurring errors. Together, these findings suggest that initial conceptual training should be complemented by continued learning through clinical use. This interpretation is consistent with previous work emphasizing that explainability and trust depend on users understanding the system’s intended purpose, outputs, and limitations [40]. Therefore, training is not “extras”; it is part of the transparency system. Well-designed training strengthens mental models so that clinicians use transparency cues for calibration (verify/override appropriately) rather than as reassurance signals that accelerate overreliance.

Attitudes, in particular, may shape the relationship between transparency and trust. Previous research has similarly recognized attitudes, perceptions, and positive expectations as factors shaping health care professionals’ trust in AI-CDSS [29]. Our findings add further context by suggesting that openness or resistance shapes whether transparency cues are attended to, dismissed, or treated as confirmation. This suggests a design requirement for plural-user transparency: systems should simultaneously (1) provide enough credibility and evidence to engage skeptics and (2) provide enough uncertainty and boundary messaging to constrain enthusiastic users from overtrust.

### Design Implications for Transparent and Trustworthy AI-CDSS

To synthesize these findings and highlight their practical implications, Table 4 summarizes key design considerations for transparent and trustworthy AI-CDSS derived from expert perspectives and provides illustrative clinical scenarios showing how these considerations could be applied in practice. Figure 2 presents a conceptual user interface mockup illustrating one possible implementation of the findings from Theme 2, while Table 5 presents a proposed training curriculum based on the interview findings.

**Table 4.** Design implications for transparent and trustworthy AI-CDSS^a^.

Theme and key insight from expert perspectives	Design implication for AI-CDSS	Illustrative clinical scenario
Theme 1: transparency and trust		
Clinicians tend to distrust “black box” systems even when performance is high.	User interface should prioritize functional transparency that supports clinical reasoning, rather than full algorithmic interpretability.	A suspected abnormality is presented with its location, confidence level, and a brief rationale.
Trust is shaped by understanding AI reasoning and limitations, not accuracy alone.	AI-CDSS should explicitly communicate uncertainty, confidence levels, and limitations rather than presenting binary or opaque outputs.	A low-confidence finding is accompanied by a verification cue and relevant model limitations.
Appropriate trust lies between overtrust and undertrust.	AI-CDSS should be designed to support trust calibration, not to maximize trust.	The clinician reviews the original radiograph before revealing the AI findings for comparison.
Theme 2: user interface		
Visual explanations (eg, heatmaps) can support interpretation but may induce automation bias.	Visual cues should be accompanied by uncertainty indicators (eg, confidence scores, caution flags) to prevent over-reliance	Each heatmap is accompanied by a finding-specific confidence score and an uncertainty cue.
Excessive information increases cognitive load and disrupts workflow.	User interface should follow a minimalist, workflow-aligned design that presents only clinically relevant information	Selecting a finding automatically displays its relevant information in a structured panel.
Misalignment with clinical workflow reduces acceptance regardless of model quality.	AI-CDSS should be seamlessly integrated into existing radiology workflows without adding unnecessary steps	AI findings are accessed within the existing image-review workflow without opening a separate application.
Expert feedback is valuable for model refinement.	AI-CDSS should enable experienced clinicians to provide feedback without increasing their workload.	Clinicians provide feedback by selecting “Agree,” “Uncertain,” or “Disagree,” with an optional comment.
Theme 3: users’ mental model		
Users require a conceptual, not technical, understanding of AI.	AI-CDSS should provide onboarding and contextual explanations about the model, training data, and performance boundaries	Brief onboarding explains the AI-CDSS’s intended use, outputs, training data, and limitations.
Misinterpretation of confidence scores or explanations can lead to misuse.	Explanatory elements should be designed to align with clinicians’ existing diagnostic reasoning practices	Confidence is described as a model estimate rather than diagnostic certainty.
Trust becomes calibrated through repeated real-case use.	AI-CDSS should support gradual learning and adaptation rather than enforcing rigid reliance	Repeated use helps clinicians recognize the AI-CDSS’s strengths and limitations across cases.
Attitudes toward AI range from resistance to over-deference.	System communication and framing should consistently position AI as a supportive tool rather than a replacement for clinical judgment	AI outputs are presented for clinician review, with the final decision remaining with the clinician.

a AI-CDSS: AI–based Clinical Decision Support System.

**Figure 2.**
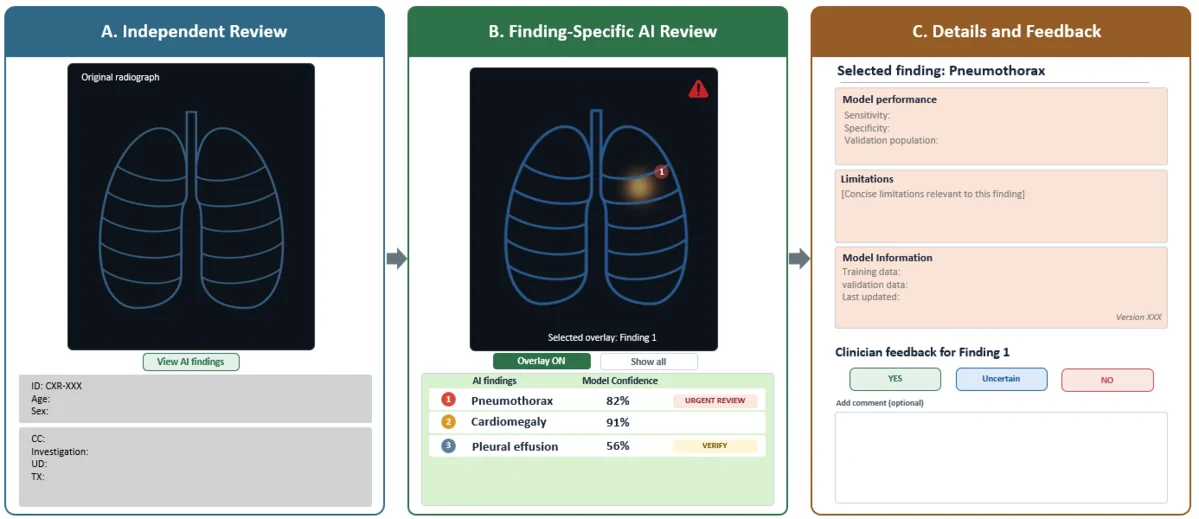
Conceptual user interface mockup for transparent communication in a chest X-ray AI-CDSS. CC: Clinical context; CDSS: Clinical decision support system; CXR: chest X-ray; TX: Treatment; UD: Underlying disease.

**Table 5.** Proposed training modules for supporting the users’ mental models of AI-CDSS.

Module	Training content
Introduction to AI and AI-CDSS^a^	Conceptual overview of how AI-CDSS generates outputs and its intended purpose and scope
System capabilities, limitations, and trust calibration	Target findings, areas of reliable and unreliable performance, common false-positive patterns, sensitivity and specificity, limitations, and the risks of undertrust and overtrust
Training data and model validation	Conceptual explanation of training data characteristics, training focus, experimental design, and model validation without unnecessary technical detail
Interpretation and practical use of AI outputs	Meaning and appropriate use of AI findings, confidence and uncertainty information, heatmaps, displayed performance metrics, findings requiring caution, and situations in which users should rely on or further verify AI outputs

a AI-CDSS: AI-based Clinical Decision Support Systems.

### Limitation and Future Work

First, this study focused on chest X-ray diagnosis; therefore, transparency and trust requirements may differ across other diagnostic contexts with varying information needs, risk levels, or interpretive workflows. Second, this study was conducted primarily in Thailand; thus, participants’ perspectives may reflect local disease patterns, including those associated with tropical settings, as well as clinical workflows, resource availability, and experience with AI-CDSS. Although context-specific preferences may not be directly transferable to all health care systems, the broader principles of understandable AI communication, uncertainty disclosure, workflow-aligned design, and support for users’ mental models may have wider relevance. Multicountry studies across diverse clinical and health care contexts are needed to assess the international applicability of these findings. Third, the purposive sample of experienced radiologists and AI experts may not capture the perspectives of novice users or clinicians without prior AI-CDSS experience. Participants’ existing experience with AI-CDSS may also have been associated with greater technology affinity, potentially underrepresenting barriers faced by less willing adopters. Fourth, the interview guide underwent researcher review, expert review, and assessment using the IOC; however, it was not formally pilot tested or cognitively pretested with representatives of the target groups. Consequently, potential differences in how participants interpreted the questions may not have been identified before data collection. Fifth, interviews shared a chest X-ray AI-CDSS context; however, the absence of a standardized scenario or visual stimulus may have led participants to refer to systems with different functionalities and user interfaces, potentially affecting their mental representations and the comparability of some responses. Future studies could use a shared prototype or standardized scenarios to provide a more consistent basis for eliciting perspectives. Finally, interviews were conducted in Thai, and quotations were reported in English. The Thai and English versions were checked against each other in collaboration with another researcher (AJW) to preserve meaning. Nevertheless, some linguistic nuances and culturally specific meanings may not have been fully conveyed in translation. Future research could adopt a mixed methods design by developing a survey instrument based on the themes identified in this study. Administering the instrument to a larger and more diverse sample of radiologists and AI experts could complement the qualitative findings, quantify the distribution of the identified attitudes and perspectives, and examine their associations with professional role, clinical experience, and prior experience with AI-CDSS.

### Conclusions

This qualitative study demonstrates that trust in AI-CDSS is fundamentally shaped by transparency as experienced through human-AI interaction, rather than by algorithmic accuracy alone. Radiologists and AI developers converged on the view that transparent communication through interpretable explanations, visible uncertainty, and clearly defined system boundaries enables clinicians to integrate AI outputs into clinical reasoning while retaining professional autonomy. Our findings further show that trust is dynamic and calibrated, developing through repeated use, feedback, and alignment between system behavior and clinicians’ mental models. Importantly, transparency must be enacted not only through interface design, but also through consideration of users’ experience, knowledge, and attitudes toward AI. Together, these results underscore that designing trustworthy AI-CDSS requires a human-centered approach, in which transparency supports appropriate reliance rather than blind acceptance or rejection. By aligning technical design with clinical workflows and users’ mental models, AI-CDSS can function as a reliable collaborative partner in real-world diagnostic practice.

## Supplementary material

10.2196/99472 Multimedia Appendix 1 Interview questions.

10.2196/99472 Multimedia Appendix 2 Interview guide.

## Abbreviations

AI-CDSS: AI-based Clinical Decision Support Systems
CTO: chief technology officer
ECG: electrocardiogram
HCI: human-computer interaction
IOC: Index of Item-Objective Congruence
RQ: research question
