# Supplementary material for: Expert Perspectives on Transparency and Trust in AI-Based Clinical Decision Support Systems for Chest X-Ray Diagnosis: Qualitative Study
Source: JMIR Med Inform. 2026 Nov 5;14:e99472. doi: 10.2196/99472 (PMC13739206; doi:10.2196/99472)
Supplement: Supplementary file 1 [file medinform-v14-e99472-s001.pdf]

Interview Questions – Radiologist

#	Question	คำถาม
1.General Questions on AI Use in Chest X-ray		
1.1	Could you describe your experience using AI-chest X-ray diagnosis? (e.g., positive/negative experiences, or any concerns)	คุณช่วยอธิบายประสบการณ์ของคุณในการใช้ระบบ AI สำหรับการวินิจฉัยภาพเอกซเรย์ทรวงอกได้หรือไม่? (เช่น ดี/ไม่ดี มีอะไรที่น่ากังวลหรือไม่)
1.2	How do you use AI output/recommendation in your diagnostic process?	คุณใช้ผลลัพธ์หรือคำแนะนำจาก AI ในกระบวนการวินิจฉัยของคุณอย่างไร?
2. Transparency and Trust for using AI-chest X-ray		
2.1	Do you think the decision-making process of AI is sufficiently transparent? (e.g., it provide relevant information about its decision-making process that helps you understand the rationale behind its output?)	คุณมองว่ากระบวนการตัดสินใจของ AI มีความโปร่งใสเพียงพอหรือไม่ (เช่น เปิดเผยข้อมูลที่เกี่ยวข้องเกี่ยวกับกระบวนการตัดสินใจ ซึ่งช่วยให้คุณเข้าใจที่มาของคำตอบ)
2.2	How do you think about the reliability of the AI chest X-ray diagnosis you use? Do you trust them? Why?	คุณคิดอย่างไรเกี่ยวกับความน่าเชื่อถือของ AI สำหรับการวินิจฉัยภาพเอกซเรย์ทรวงอกที่คุณใช้? คุณไว้วางใจระบบเหล่านี้หรือไม่? ทำไม?
2.3	Does the system's ability to explain its decisions affect your level of trust? If so, what information would you like the system to provide to help you understand the reasoning behind its decisions?	ความสามารถในการอธิบายเหตุผลในตัดสินใจของระบบ AI มีผลต่อระดับความไว้วางใจของคุณหรือไม่? หากเป็นเช่นนั้น คุณต้องการให้ระบบให้ข้อมูลใดเพื่อช่วยให้คุณเข้าใจเหตุผลในการตัดสินใจของระบบ?
2.4	Are you more likely to trust AI in some cases than others? Why?	คุณมีแนวโน้มที่จะไว้วางใจ AI มากขึ้นในบางกรณีมากกว่ากรณีอื่น ๆ หรือไม่? เพราะเหตุใด?
3. User Interface (UI)		
3.1	How do you interact with the AI chest X-ray user interface? Which UI features do you use for diagnostics?	คุณมีปฏิสัมพันธ์กับส่วนเชื่อมต่อผู้ใช้ของ AI อย่างไร? คุณใช้ฟีเจอร์ลักษณะใดบนหน้าจอสำหรับการวินิจฉัย?
3.2	What kind of information would you like the system to provide to help you decide whether to trust it or not? In the current system, which UI features provide that information?	ข้อมูลใดที่คุณใช้เพื่อช่วยในการตัดสินที่จะไว้วางใจผลลัพธ์ของ AI? และไม่ไว้วางใจ? ฟีเจอร์บนหน้าจอใดที่แสดงข้อมูลนั้น?
3.3	Would you prefer only one AI output, or would you like additional data to support the decision? (e.g., multiple possible answers with confidence scores, or results with model performance)	คุณต้องการผลลัพธ์ของ AI เพียงผลลัทธ์เดียวหรือไม่? หรือต้องการข้อมูลเพื่อเติม (เช่น ต้องการคำตอบหลายข้อแสดงพร้อมความเชื่อมั่น หรือผลลัพธ์พร้อมประสิทธิภาพโมเดล)
3.4	What visual elements help you better understand AI outputs?	องค์ประกอบบนภาพใดที่ช่วยให้คุณเข้าใจผลลัพธ์ของ AI ได้ดีขึ้น?
3.5	Does the way information is presented affect the effectiveness of AI systems? Does it influence the perception of transparency or trust?	รูปแบบการนำเสนอข้อมูล มีผลต่อการใช้งานระบบ AI อย่างมีประสิทธิภาพหรือไม่? ส่งผลกับการรับรู้ความโปร่งใสหรือความไว้วางใจหรือไม่?

#	Question	คำถาม
3.6	Would you like the UI to be customizable (e.g., allowing you to prioritize or display only the information you need)? Additionally, would you want to provide feedback to the system? What specific features would you want to see in the system to facilitate this interaction?	คุณต้องการให้ส่วนเชื่อมต่อผู้ใช้ปรับแต่งได้หรือไม่ (เช่น ให้คุณกำหนดลำดับความสำคัญหรือแสดงเฉพาะข้อมูลที่คุณต้องการ) นอกจากนี้ คุณต้องการให้ข้อเสนอแนะกับระบบหรือไม่ คุณต้องการเห็นคุณลักษณะเฉพาะใดบ้างในระบบเพื่ออำนวยความสะดวกในการโต้ตอบนี้
3.7	Are there any other factors you would recommend for user interaction?	คุณมีปัจจัยใดสำหรับส่วนเชื่อมต่อผู้ใช้ที่ต้องการแนะนำเพิ่มเติมหรือไม่ ?
4. Users' Mental Model		
4.1	Do you think a clinician's level of expertise or years of experience affects their decision to use or trust the system? How?	คุณคิดว่าความเชี่ยวชาญหรืออายุงานของแพทย์ มีผลต่อการตัดสินใจใช้ระบบหรือไว้วางใจในระบบหรือไม่? อย่างไร?
4.2	Do you think personal attitudes or biases affect trust in the system? What are those attitudes or biases? (e.g., previous experience with AI, resistance to using AI systems, or confidence in own judgment) What strategies do you think could help mitigate these biases?	คุณคิดว่าทัศนคติหรืออคติส่วนตัวส่งผลกระทบต่อการไว้วางในในระบบหรือไม่? ทัศนคติหรืออคตินั้นคืออะไร? (เช่น ประสบการณ์ใช้ AI ก่อนหน้า ความต่อต้านการใช้งานระบบ AI หรือการเชื่อมั่นในตัวเอง) คุณคิดว่ากลยุทธ์ใดที่จะช่วยลดอคติเหล่านี้ได้?
4.3	Do you think basic knowledge of AI is necessary for using the system? If so, what kind of knowledge is required (in general)? Does having basic knowledge impact the perception of transparency or trust in the system?	คุณคิดว่าในการใช้งานระบบจำเป็นความรู้พื้นฐานเกี่ยวกับระบบ AI หรือไม่? ความรู้นั้นคืออะไรบ้าง (โดยทั่วไป)? ความรู้พื้นฐานส่งผลต่อการรับรู้ความโปร่งใสหรือความไว้วางใจในระบบหรือไม่?
4.4	Are there any other factors you would recommend for user's mental models?	คุณมีปัจจัยใดสำหรับแบบจำลองความคิดของผู้ใช้งานที่ต้องการแนะนำเพิ่มเติมหรือไม่ ?

Interview Questions – AI Experts

#	Question	คำถาม
1.General Questions on AI Chest X-ray		
1.1	What are the key goals when develop AI chest X-ray diagnosis?	เป้าหมายหลักในการพัฒนาระบบ AI สำหรับการวินิจฉัยภาพเอกซเรย์ทรวงอก
2.Transparency and Trust of using AI-chest X-ray		
2.1	How do you define transparency in AI chest X-ray diagnosis?	คุณให้คำนิยามของ "ความโปร่งใส" ในระบบ AI สำหรับการวินิจฉัยภาพเอกซเรย์ทรวงอกอย่างไร?
2.2	What design element do you consider to ensure that AI output is transparent?	องค์ประกอบใดสำหรับการออกแบบที่คุณใช้เพื่อให้แน่ใจว่าผลลัทธ์ของ AI มีความโปร่งใส?
2.3	What factors of AI systems influence clinicians' trust? Which factor do you believe has the most significant, and why?	ปัจจัยใดของระบบ AI ที่มีอิทธิพลต่อความไว้วางใจของแพทย์? คุณคิดว่าปัจจัยใดสำคัญที่สุด เนื่องจากสาเหตุใด?
2.4	How do you design AI systems to prevent over-trust or under-trust from clinicians?	คุณออกแบบระบบ AI อย่างไรเพื่อลดปัญหาความไว้วางใจมากเกินไป (Overtrust) หรือความไว้วางใจน้อยเกินไป (Undertrust) จากแพทย์?
2.5	What are the current limitations or challenges that prevent AI output from being explained clearly?	มีข้อจำกัดหรือความท้าทายอะไรบ้างในปัจจุบัน ที่ทำให้การอธิบายผลลัพธ์ของ AI ชัดเจนไม่เพียงพอ?
3. User Interface		
3.1	What information do you think clinicians should know from the AI chest X-ray system during diagnosis? What types of information should be provided, and in what form?	คุณคิดว่าแพทย์ควรทราบข้อมูลใดบ้างจากระบบเอกซเรย์ทรวงอก AI ในระหว่างการวินิจฉัย? ข้อมูลอะไรบ้างและมีลักษณะใด?
3.2	What user interface factor do you use to make AI output explainable to clinicians? Which feature show that factor?	ปัจจัยใดในส่วนเชื่อมต่อผู้ใช้ที่คุณใช้เพื่อทำให้ผลลัพธ์ของ AI มีความสามารถในการอธิบายแก่แพทย์ได้? ฟีเจอร์ใดแสดงถึงปัจจัยดังกล่าว?
3.3	What user interface factor influence whether clinicians perceive AI output as reliability or unreliability? Which feature show that factor?	ปัจจัยใดของส่วนเชื่อมต่อผู้ใช้ที่มีอิทธิพลต่อการรับรู้ของแพทย์ว่า AI มีความน่าเชื่อถือหรือไม่น่าเชื่อถือ? ฟีเจอร์ใดแสดงถึงปัจจัยดังกล่าว?
3.4	If the information must be limited to avoid overload and you can choose only three key details to display on the user interface, what should be presented to clinicians?	หากต้องจำกัดข้อมูลเพื่อหลีกเลี่ยงการรับรู้ข้อมูลที่มากเกินไป และคุณสามารถเลือกได้เพียงรายละเอียดสำคัญสามประการที่จะแสดงบนส่วนเชื่อมต่อผู้ใช้ อะไรที่ควรถูกนำเสนอให้กับแพทย์?
3.5	Do different formats of AI output impact clinicians' trust and reliance on AI output? If so, which factor do you consider the most important?	รูปแบบผลลัพธ์ของ AI ที่แตกต่างกันส่งผลกระทบต่อความไว้วางใจและการพึ่งพาผลลัพธ์ของ AI ของแพทย์หรือไม่ หากเป็นเช่นนั้น คุณคิดว่าปัจจัยใดสำคัญที่สุด
3.6	Would you like the UI to be customizable (e.g., allowing user to prioritize or display only the information user's need)? Additionally, would you want users to provide feedback to the system? What specific features are you think to facilitate this interaction?	คุณต้องการให้ UI ปรับแต่งได้หรือไม่ (เช่น อนุญาตให้ผู้ใช้กำหนดลำดับความสำคัญหรือแสดงเฉพาะข้อมูลที่ผู้ใช้ต้องการ)? นอกจากนี้ คุณต้องการให้ผู้ใช้ให้ข้อเสนอแนะกับระบบหรือไม่ คุณคิดว่ามีคุณลักษณะเฉพาะใดบ้างที่จะช่วยอำนวยความสะดวกในการโต้ตอบนี้?
3.7	Are there any other factors you would recommend for user interaction?	คุณมีปัจจัยใดสำหรับส่วนเชื่อมต่อผู้ใช้ที่ต้องการแนะนำเพิ่มเติมหรือไม่?

#	Question	คำถาม
4. User's Mental Models		
4.1	Do you think clinicians' attitudes toward AI or experience impact how they interpret AI output? How?	คุณคิดว่าทัศนคติของแพทย์ที่มีต่อ AI หรือประสบการณ์ มีผลต่อวิธีที่พวกเขาตีความผลลัพธ์จาก AI หรือไม่? อย่างไร?
4.2	What strategies can help mitigate clinician bias in the role of AI in clinical decision-making?	มีกลยุทธ์ใดบ้างที่สามารถช่วยลดอคติของแพทย์ต่อบทบาทของ AI ในกระบวนการตัดสินใจทางคลินิก?
4.3	What are the key gaps between AI developers' expectations and how clinicians perceive or interact with AI systems?	อะไรคือช่องว่างที่สำคัญระหว่างความคาดหวังของนักพัฒนา AI และวิธีที่แพทย์รับรู้หรือโต้ตอบกับระบบ AI?
4.4	Do you think basic knowledge of AI is necessary for using the system? If so, what kind of knowledge is required (in general)? Does having basic knowledge impact the perception of transparency or trust in the system?	คุณคิดว่าในการใช้งานระบบจำเป็นความรู้พื้นฐานเกี่ยวกับระบบ AI หรือไม่? ความรู้นั้นคืออะไรบ้าง (โดยทั่วไป)? ความรู้พื้นฐานส่งผลต่อการรับรู้ความโปร่งใสหรือความไว้วางใจในระบบหรือไม่?
4.5	Are there any other factors you would recommend for user's mental models?	คุณมีปัจจัยใดสำหรับแบบจำลองความคิดของผู้ใช้งานที่ต้องการแนะนำเพิ่มเติมหรือไม่ ?
