# Supplementary material for: Expert Perspectives on Transparency and Trust in AI-Based Clinical Decision Support Systems for Chest X-Ray Diagnosis: Qualitative Study
Source: JMIR Med Inform. 2026 Nov 5;14:e99472. doi: 10.2196/99472 (PMC13739206; doi:10.2196/99472)
Supplement: Supplementary file 2 [file medinform-v14-e99472-s002.pdf]

Participant Briefing Slides: Thai Original

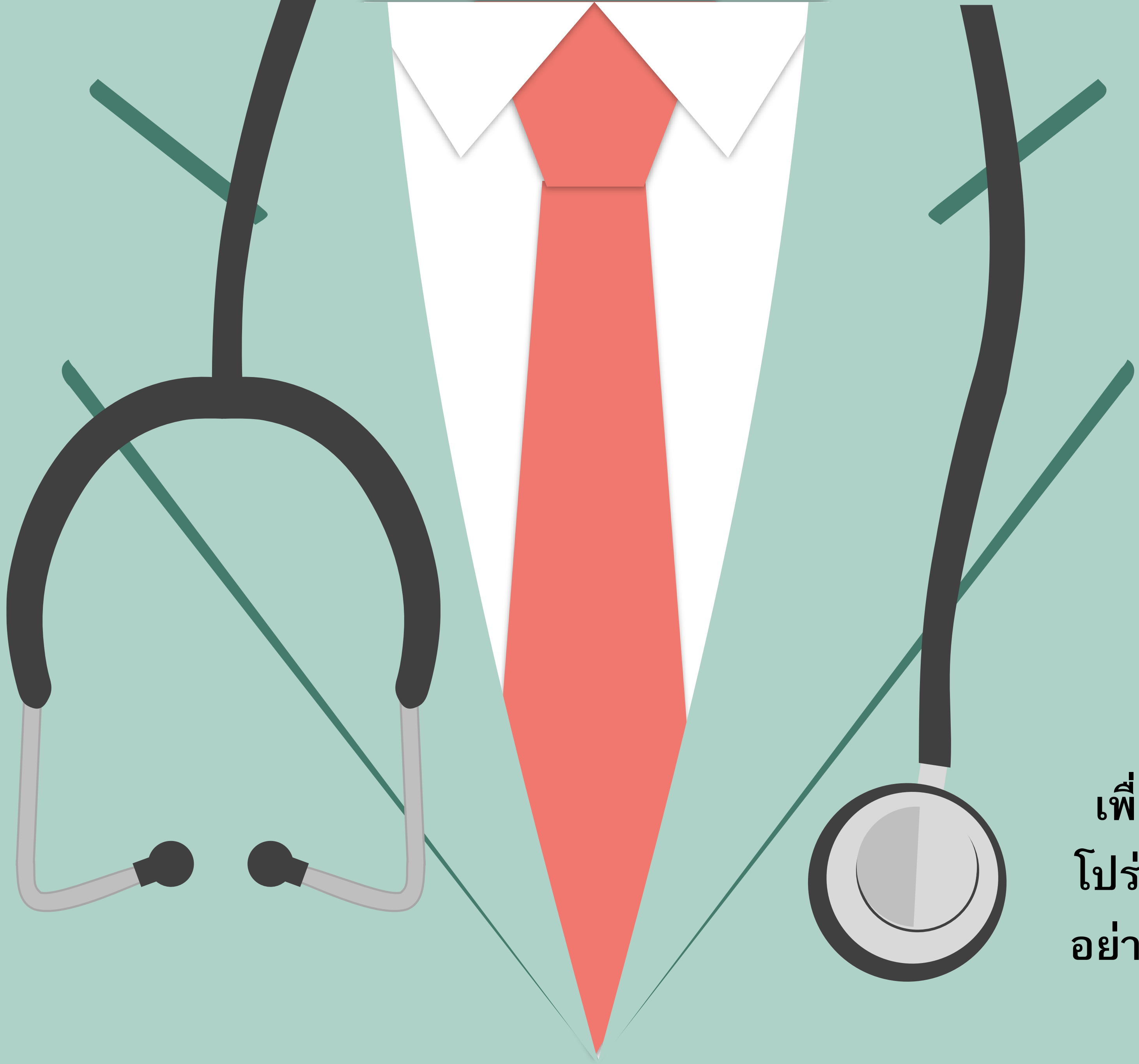

การสัมภาษณ์ผู้เชี่ยวชาญ
เพื่อระบุองค์ประกอบที่สนับสนุนการสื่อสารที่
โปร่งใสสำหรับปฏิสัมพันธ์ระหว่างมนุษย์และ AI
อย่างมีประสิทธิผลกรณีศึกษา: ภาพรังสีทรวงอก

ภาพรวมการสัมภาษณ์

(20 - 30 นาที)

อธิบายวัตถุประสงค์งานวิจัย

ตอบแบบสอบถามเชิงประชากร

เริ่มสัมภาษณ์

สิ้นสุดการสัมภาษณ์

ภาพรวมการใช้งานระบบ

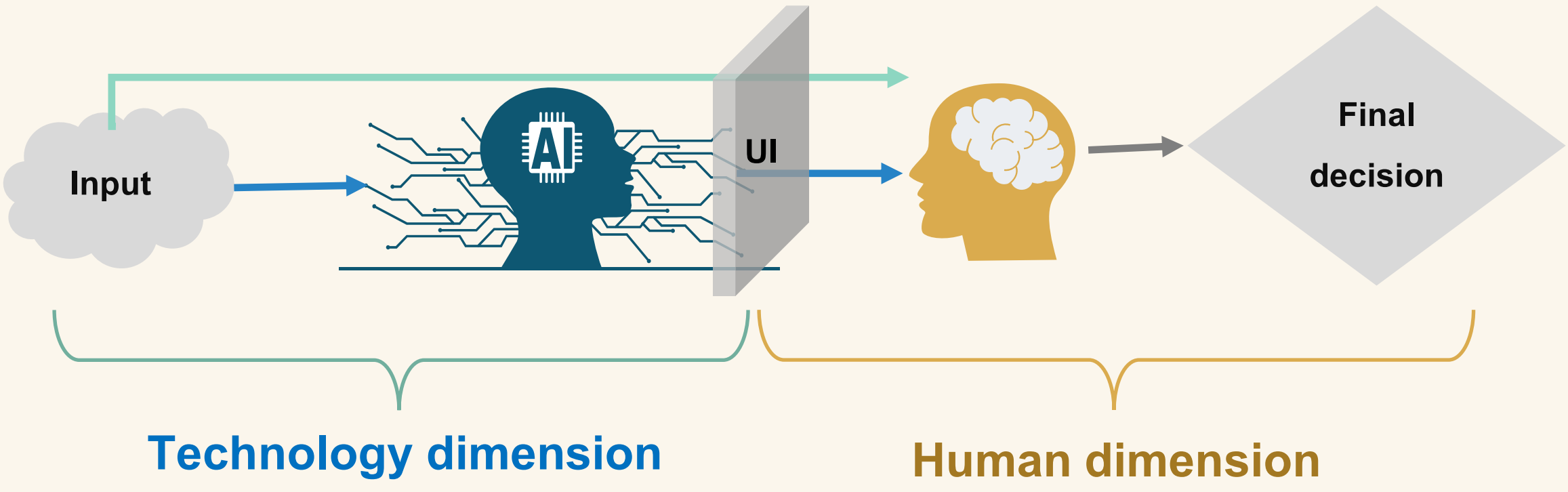

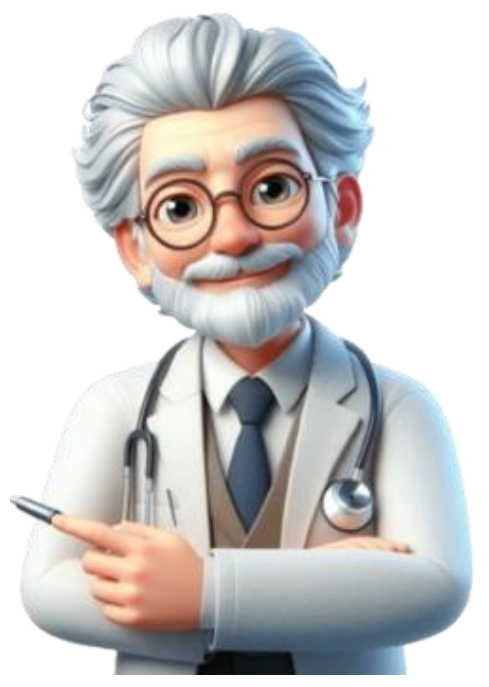

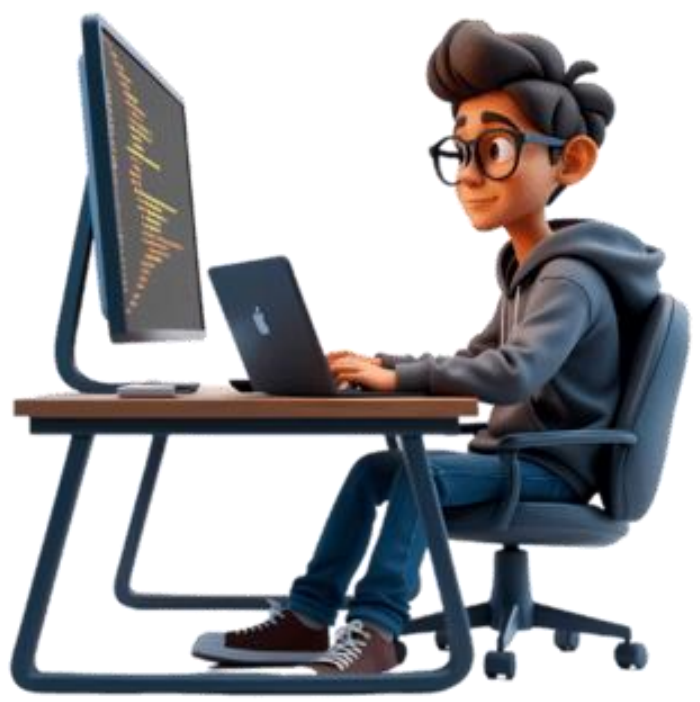

วัตถุประสงค์ของการสัมภาษณ์

➢ **เพื่อระบุองค์ประกอบสำหรับสนับสนุนการสื่อสารที่โปร่งใสสำหรับปฏิสัมพันธ์ระหว่างมนุษย์และ AI อย่างมีประสิทธิผล โดยมุ่งเน้นที่หน้าจอและแบบจำลองทางจิตของผู้ใช้งาน: กรณีศึกษา ภาพรังสีทรวงอก**

ความโปร่งใส: การเปิดเผยข้อมูลที่เกี่ยวข้องเกี่ยวกับกระบวนการตัดสินใจ ขั้นตอน ประสิทธิภาพ และการทำงานของระบบ

ความไว้วางใจ: ความเชื่อว่าระบบมีความสามารถ ทักษะ และความเชี่ยวชาญในการทำหน้าที่เพื่อบรรลุผลประโยชน์สูงสุดที่ต้องการ

หน้าจอ: ส่วนของระบบที่ผู้ใช้ในการรับข้อมูลและโต้ตอบกับระบบ

แบบจำลองความคิดของผู้ใช้: ความรู้และความเข้าใจของแต่ละบุคคล ซึ่งเป็นพื้นฐานในการตีความและใช้งานระบบใดๆ

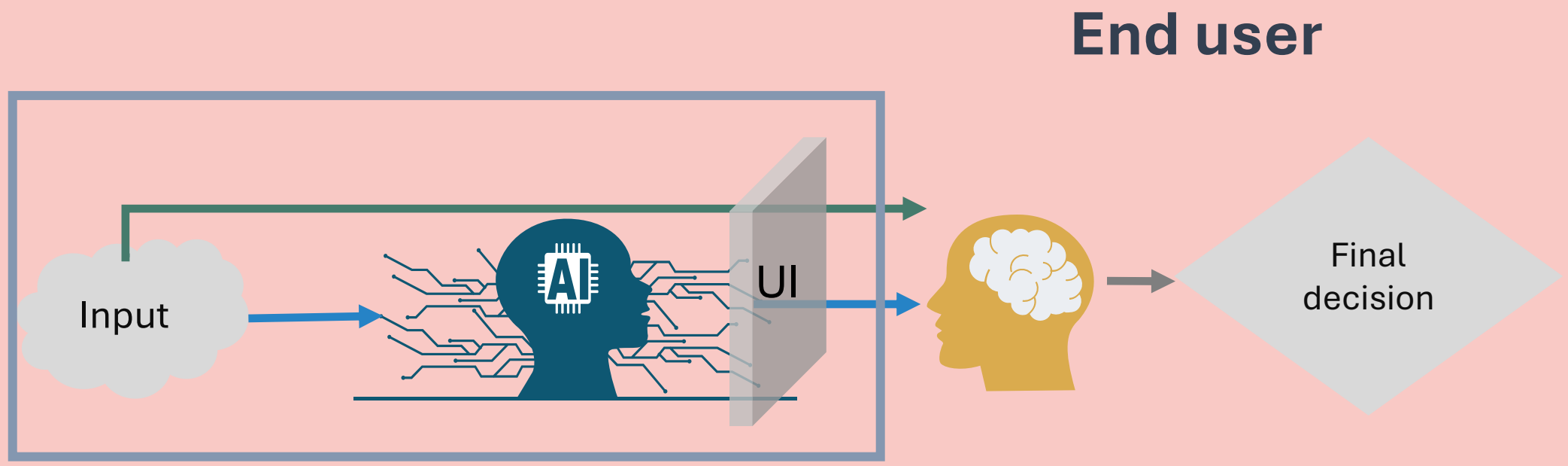

รูปที่ 1 ภาพรวมการมีปฏิสัมพันธ์ระหว่างมนุษย์และAI

ประเด็นการสัมภาษณ์

I. คำถามทั่วไปเกี่ยวกับการใช้ AI สำหรับการวินิจฉัยภาพเอกซเรย์ทรวงอก (General Questions on AI-CDSS for Chest X-ray)

II. ความโปร่งใสและความไว้วางใจในการใช้ AI สำหรับการวินิจฉัยภาพเอกซเรย์ทรวงอก (Transparency and Trust in AI-CDSS for Chest X-ray)

III. การใช้งานหน้าจอ (User Interface)

IV. แบบจำลองทางจิตของผู้ใช้งาน (Users' Mental Models)

- ขออนุญาตอัดเสียง
- ลบภายใน 30 วัน นับจากวันสัมภาษณ์
- ข้อมูลเป็นความลับ ไม่มีการเปิดเผยตัวตน
- ข้อมูลที่ได้จะถูกนำไปถอดเสียงสัมภาษณ์เพื่อทำการวิเคราะห์
- การสัมภาษณ์จะถูกเปิดเผยในรูปแบบของการสรุปผลวิจัยเท่านั้น

- สามารถขอหยุด เลิกให้สัมภาษณ์ หรือบอกเลิกการเข้าร่วมโครงการวิจัยเมื่อใดก็ได้

แบบสอบถามเชิงประชากร

การสัมภาษณ์ผู้เชี่ยวชาญ

I. คำถามทั่วไปเกี่ยวกับการใช้ AI สำหรับการวินิจฉัยภาพเอกซเรย์ทรวงอก

(General Questions on AI-CDSS for Chest X-ray)

การสัมภาษณ์ผู้เชี่ยวชาญ

II. ความโปร่งใสและความไว้วางใจในการใช้ AI สำหรับการวินิจฉัยภาพเอกซเรย์ทรวงอก

(Transparency and Trust in AI-CDSS for Chest X-ray)

การสัมภาษณ์ผู้เชี่ยวชาญ

III. การใช้งานหน้าจอ

(User Interface)

การสัมภาษณ์ผู้เชี่ยวชาญ

VI. แบบจำลองทางจิตของผู้ใช้งาน

(Users’ Mental Models)

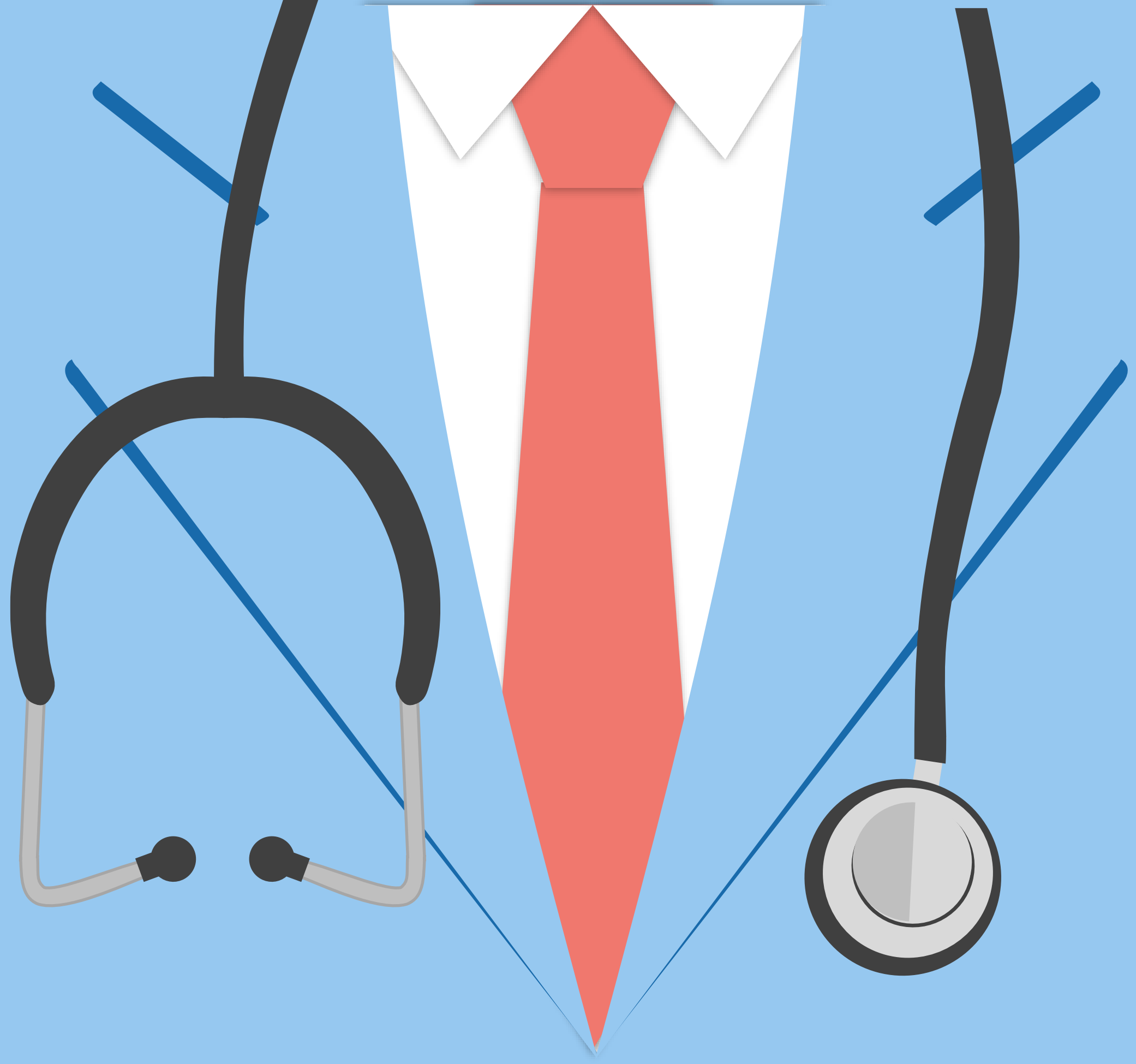

THANK YOU

Participant Briefing Slides: English Translation

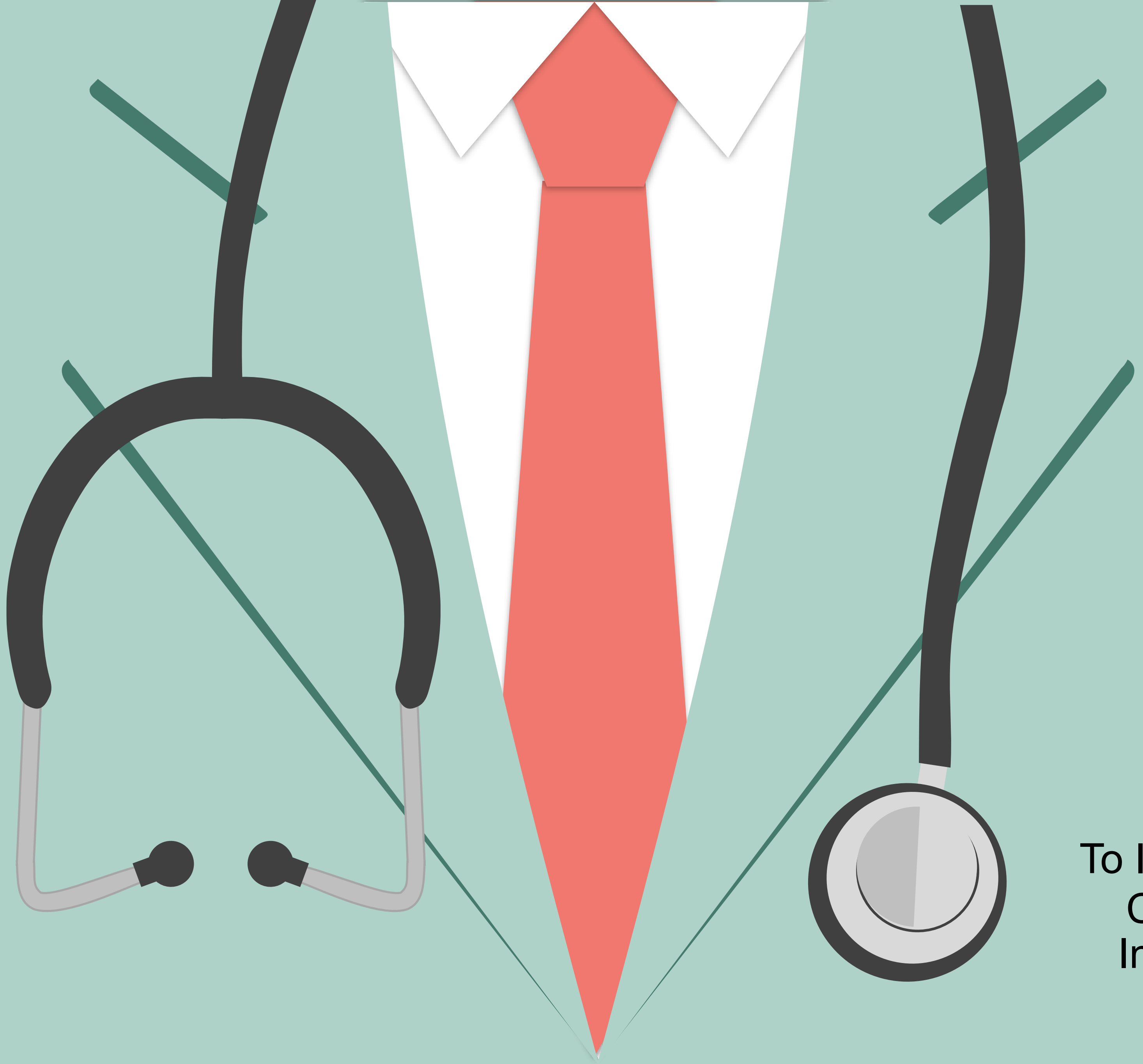

Expert Interviews

To Identify Elements Supporting Transparent Communication for Effective Human–AI Interaction: A Case Study of Chest X-ray

ภาพรวมการสัมภาษณ์

(20 - 30 นาที)

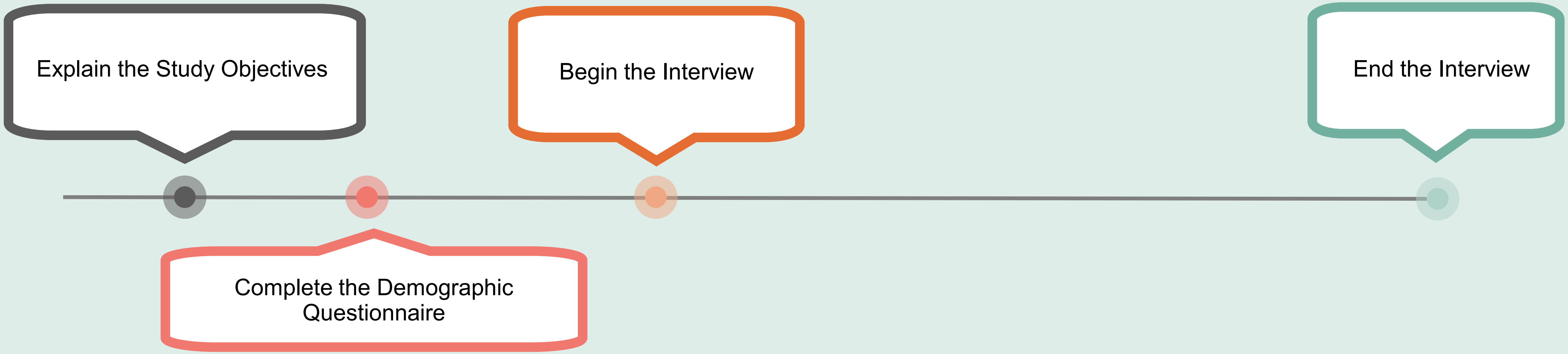

Overview of Human–AI Interaction

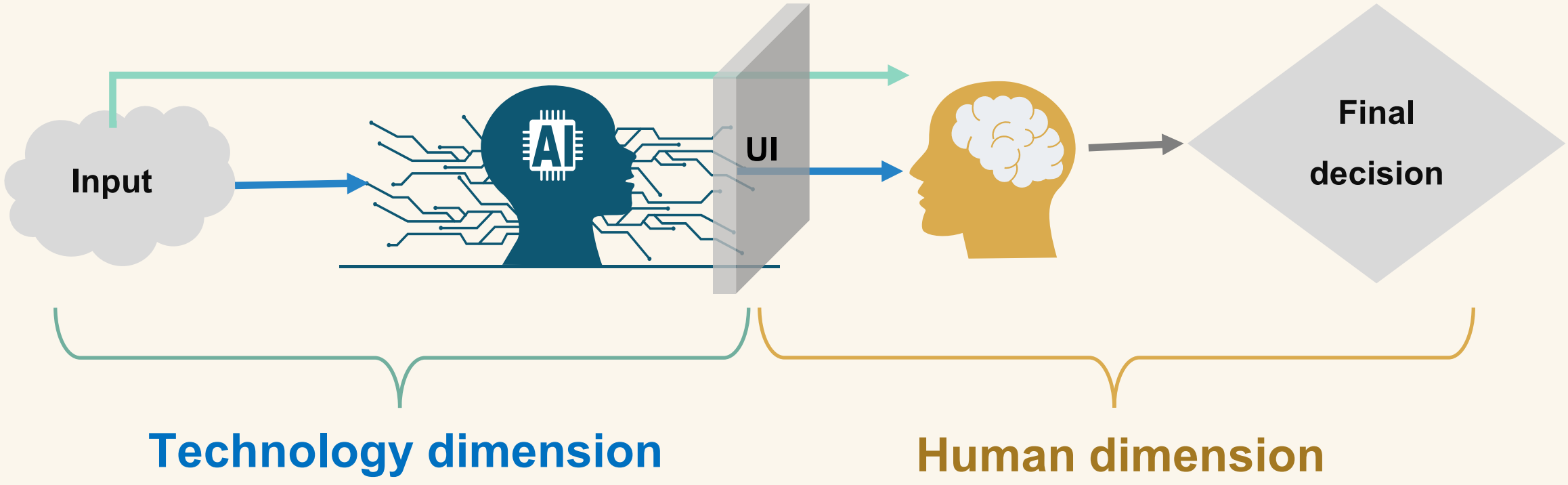

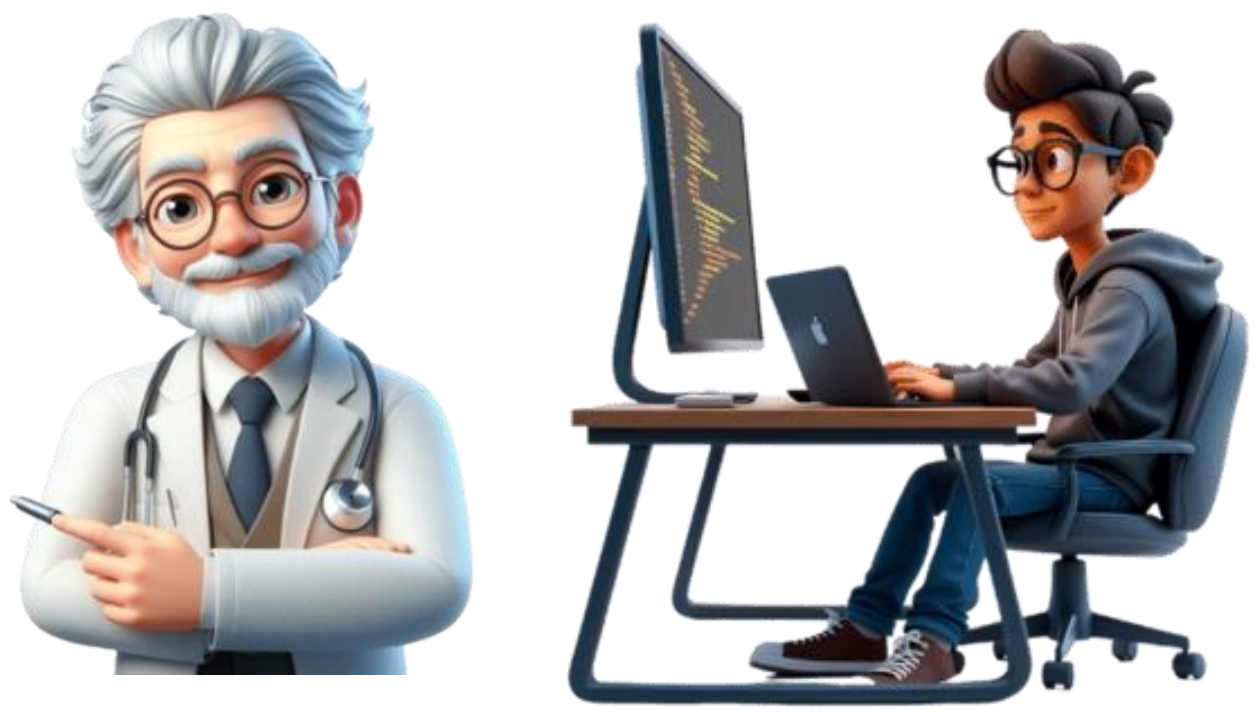

วัตถุประสงค์ของการสัมภาษณ์

- **เพื่อระบุองค์ประกอบสำหรับสนับสนุนการสื่อสารที่โปร่งใสสำหรับปฏิสัมพันธ์ระหว่างมนุษย์และ AI อย่างมีประสิทธิผล โดยมุ่งเน้นที่หน้าจอและแบบจำลองทางจิตของผู้ใช้งาน: กรณีศึกษา ภาพรังสีทรวงอก**

Transparency: the disclosure of relevant information about a system's decision-making processes, procedures, performance, and functioning.

Trust: the belief that a system has the capability, skills, and expertise to perform its functions to achieve the maximum desired benefits.

User interface: the part of a system through which users receive information and interact with the system

Users' mental model: an individual's knowledge and understanding that form the basis for interpreting and using a system.

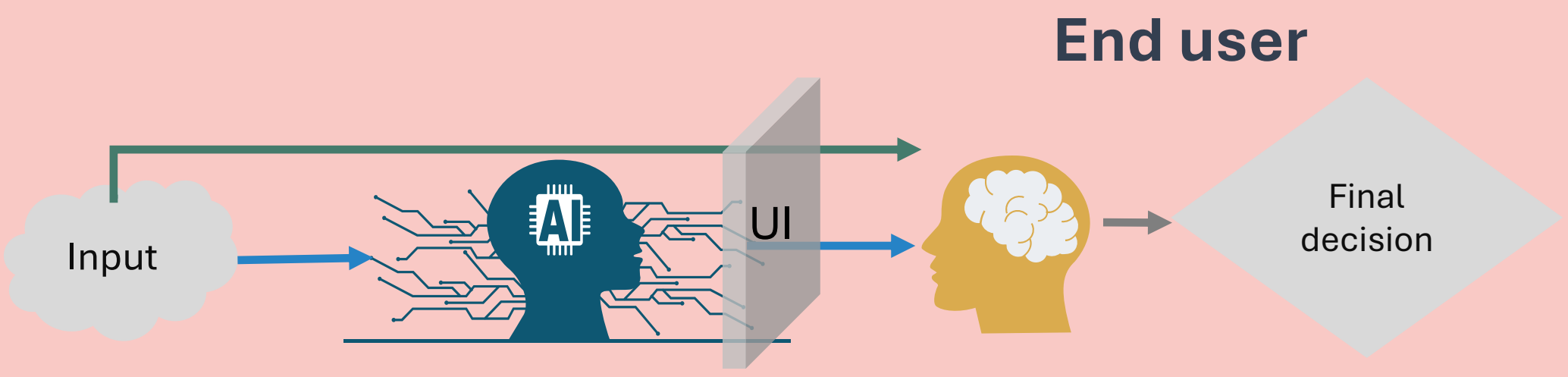

Figure 1 Overview of Human–AI Interaction

Topic of interview

I. General Questions on AI-CDSS for Chest X-ray
II. Transparency and Trust in AI-CDSS for Chest X-ray
III. User Interface
IV. Users' Mental Models

- Permission is requested to audio-record the interview.
- Audio recordings will be deleted within 30 days of the interview.
- The data will remain confidential. Participants' identities will not be disclosed.
- Interview recordings will be transcribed for analysis.
- Interview findings will be reported only in summarized form.

- You may request a pause, end the interview, or withdraw from the study at any time.

Demographic Questionnaire

Expert Interviews

I. General Questions on AI-CDSS for Chest X-ray

Expert Interviews

II. Transparency and Trust in AI-CDSS for Chest X-ray

Expert Interviews

III. User Interface

Expert Interviews

VI. Users’ Mental Models

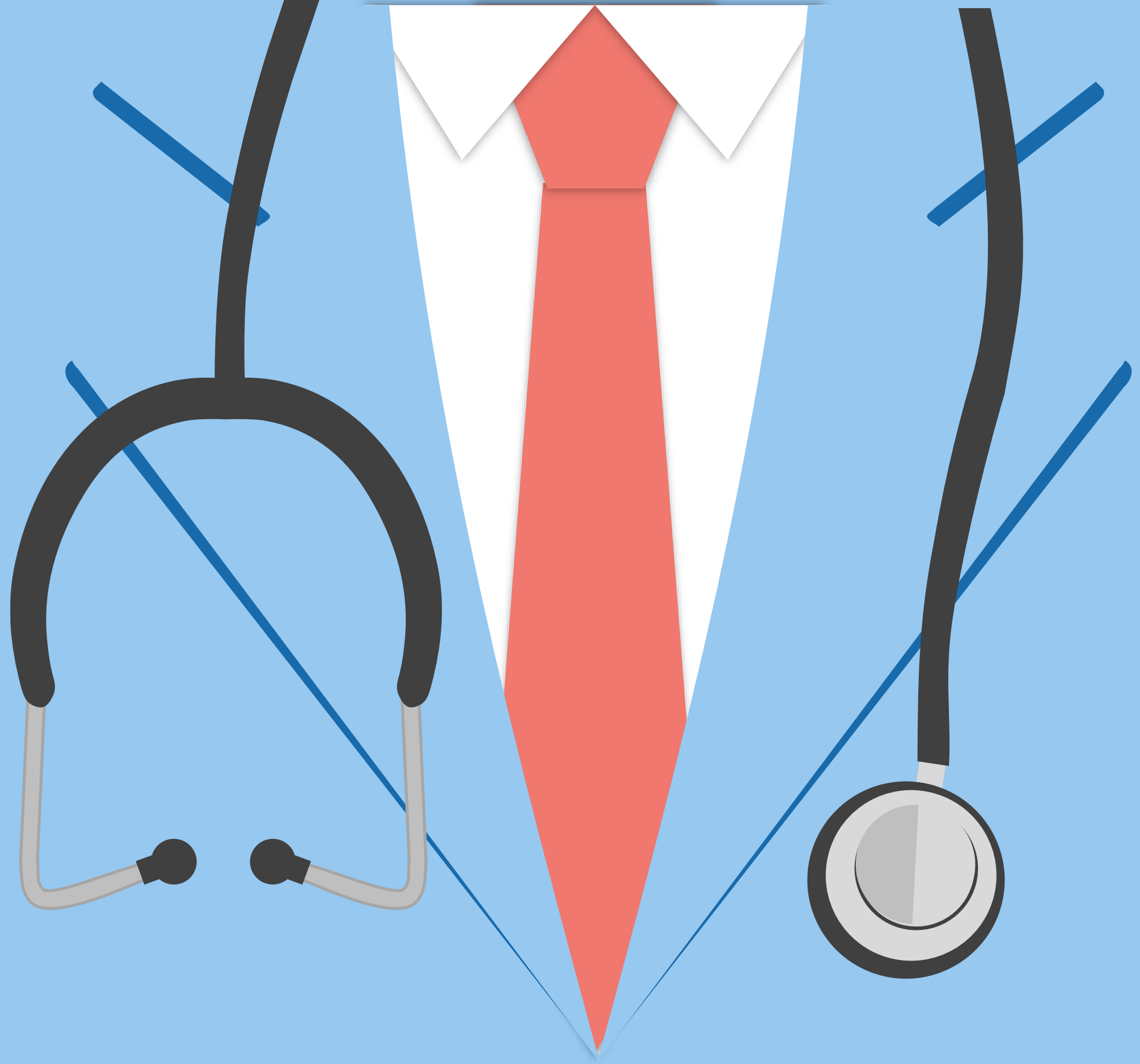

THANK YOU
